# Deciphering the Sodium Sensing Mechanisms in Glycophytes and Halophytes

**DOI:** 10.1111/pce.70128

**Published:** 2025-09-01

**Authors:** Rabia Areej Cheema, Hafiz Mamoon Rehman, Sehar Nawaz, Shakeel Ahmad, Hon‐Ming Lam

**Affiliations:** ^1^ Centre of Agricultural Biochemistry and Biotechnology University of Agriculture Faisalabad Faisalabad Pakistan; ^2^ School of Life Sciences and Center for Soybean Research of the State Key Laboratory of Agrobiotechnology The Chinese University of Hong Kong Shatin China; ^3^ Seed Center and Plant Genetic Resources Bank, Ministry of Environment Water and Agriculture Riyadh Saudi Arabia

**Keywords:** cyclic nucleotide‐gated channel (CNGC), glutamate receptor (GLR), glycophyte, glycosyl inositol phosphoryl ceramide (GIPC), halophyte, nonselective cation channel (NSCC), receptor‐like kinase (RLK), sodium ion sensing

## Abstract

Plants, including halophytes (salt‐tolerant) and glycophytes (salt‐sensitive), have developed diverse molecular mechanisms and morphological adaptations to survive in saline environments. The cellular components and molecular processes for salinity sensing and stress tolerance have been extensively identified in glycophytes, but not so with halophytes. Salinity sensing requires the perception of a major soil salinity contributor, that is, sodium ions (Na^+^). The exact molecular mechanism or pathway for Na^+^ perception is still unclear. The investigations into potential Na^+^ sensor candidates uncovered glycosyl inositol phosphoryl ceramide (GIPC) phospholipids with direct evidence. In cells, Na^+^ ions are also sensed by various Non‐selective cation channels (NSCCs), including the cyclic nucleotide‐gated channels (CNGCs) and glutamate receptors (GLRs), and other receptor‐like kinases (RLKs). This review surveyed the roles of GIPCs, CNGCs, GLRs, RLKs, including the *Catharanthus roseus* RLK1‐like kinases, leucine‐rich repeat extensins, lectin RLKs, and wall‐associated kinases, as potential Na^+^ sensors in glycophytes and halophytes. Based on current information on these receptors, we proposed new models of Na^+^ sensing mechanisms in both plant types. The comparison of possible Na^+^ sensing mechanisms between glycophytes and halophytes might provide future research avenues for improving salt tolerance in crops.

## Summary statement

• This review compares Na^+^ perception mechanisms in glycophytes and halophytes, highlighting the key molecular candidates, including GIPCs, NSCCs, and receptor‐like kinases sensing Na^+.^
• Understanding sensing mechanisms will provide future avenues for improved salt tolerance of crop plants worldwide.

## 1 Introduction

Soil salinization is an adverse phenomenon that has devastating effects on crop production around the globe, and saline soil accounts for 7% of the world's total land area, endangering food security (Balasubramaniam et al. 2023). It was projected that, by 2050, more than 50% of the world's soil will be salinized (He et al. 2023). Researchers defined saline soil as having an electrical conductivity in the soil solution of 4 dS m^−1^ or more (Munns and Tester 2008). Soil salinity is generally classified into sodic and saline soils. Saline soils have varying amounts of different salt types, including sulfates, bicarbonates, and carbonates, for example, Na_2_CO_3_, NaHCO_3_, Na_2_SO_4_ and MgSO_4_, while sodic soils consist mainly of higher NaCl concentrations. Experts approximated the total area of land affected by salinity to be close to 1.125 billion hectares (Wicke et al. 2020).

Salt tolerance in plants exists in a continuous spectrum between two extremes, salt‐sensitive glycophytes and salt‐loving/salt‐tolerant halophytes. Glycophytes are salt‐sensitive plants that fail to complete their life cycle beyond 100 mM NaCl (Munns and Tester 2008), for example, *Arabidopsis thaliana, Oryza sativa*, and *Glycine max*, while halophytes are salt‐tolerant plants that typically thrive in ≥ 200–500 mM NaCl (Meng et al. 2018), such as *Thellungiella halophila* (salt cress), *Suaeda maritima* (herbaceous seepweed), *Avicennia marina* (grey mangrove), *Mesembryanthemum crystallinum(ice plant), Zostera marina* (eelgrass) (Volkov and Flowers 2019). Based on their affinity for salts, halophytes can be further classified into obligate halophytes, which require a saline environment for optimal growth, versus facultative halophytes, which can thrive in both nonsaline and saline conditions (Volkov and Flowers 2019).

Halophytes are broadly categorized, based on their salt management strategies, into salt accumulators (euhalophytes), salt excluders (pseudohalophytes), and salt excretors (recretohalophytes) (Meng et al. 2018), possessing morpho‐physiological abilities that are absent in glycophytes. Euhalophytes, for example, *Salicornia europaea* and *Suaeda salsa*, sequester Na^+^ and Cl^−^ in large storage vacuoles present in their succulent stems and leaves to maintain hydration and osmotic pressure (Meng et al. 2018). Pseudohalophytes, for example, mangroves such as *Avicennia marina* and the salt grass *Aeluropus* spp, prevent nearly 90% of Na^+^ from entering the roots via hydrophobic barriers such as extensive Casparian bands and suberin lamellae in the root endodermis (Volkov and Flowers 2019), and consequently < 10% of external Na^+^ reaches the shoots in mangroves (Krishnamurthy et al. 2014). On the other hand, recretohalophytes, for example, saltbushes (*Atriplex* spp) and sea lavender (*Limonium bicolor*), actively store or secrete Na^+^ via specialized epidermal structures such as salt glands or bladder hairs on leaves (Meng et al. 2018). The Na^+^ and Cl^−^ ions are moved from mesophyll cells into the bladder or gland lumen via ATP‐driven pumps, from which the salts are either washed away or crystallized on the outer surface (Faraday and Thomson 1986).

The salt‐accumulating euhalophytes sequester Na^+^ in their vacuoles via V‐ATPases and V‐PPases to generate H^+^ gradients via vacuolar Na^+^/H^+^ antiporters (NHXs), incurring high energy costs in the process. (Munns et al. 2020; Melino and Tester 2023). However, they offset this energy cost by upregulating V‐ATPases and V‐PPases, using Na^+^ as a cheap osmoticum, and minimizing the use of costly organic osmolytes favored by glycophytes (Gao et al. 2011). Sodium excluders limit Na^+^ influx and extrude Na^+^ from roots via energy‐consuming plasma‐membrane H^+^‐ATPases and SOS1‐type NHXs. Halophytes optimize their energy use by remodeling their root architecture and reducing Na^+^ influx, as opposed to permitting high Na^+^ influx followed by energy‐consuming Na^+^ efflux, like in glycophytes (Bojórquez‐Quintal et al. 2014).

All plants, whether they be glycophytes or halophytes, sense and import salt in ionic form upon initial salt exposure (Van Zelm et al. 2020). Glycophytes are extremely vulnerable to Na^+^‐induced damage and will eventually die as a result. Therefore, it is crucial to determine Na^+^ sensing pathways and mitigate the harmful effects of excess Na^+^ in plants. The comparison between the putative Na^+^ sensing strategies of halophytes and glycophytes will add to our current knowledge and help with enhancing crop salt tolerance (Van Zelm et al. 2020). Previous studies define a Na^+^ sensor as a molecule or protein that perceives variations in the concentration of extracellular Na^+^ and transmits this information to the interior of the cell. The first step in salt stress responses of plants involves the sensing and perception of ions, most commonly Na^+^ (Maathuis 2014). The information regarding the initial Na^+^ sensing is limited in plants. Various channels, transporters, and putative molecules have been hypothesized as potential Na^+^ sensors and are listed in Table 1.

**Table 1:** List of potential or possible sodium (Na^+^) sensors in plants.

Potential Na^+^ sensor	Locations (cellular/subcellular)	Examples	Plant species	Roles in Na^+^ homeostasis or functions in cell	References
Dual‐affinity high‐affinity potassium transporters (HKTs)	Roots and shoots, cell membrane	HKT1 family (low‐affinity Na^+^‐selective transport), HKT2 family (symporter for Na^+^ and K^+^ ions)	*Triticum monococcum* and *Triticum aestivum*	Dual‐affinity HKTs transport Na^+^ as well as K^+^. These contribute to the exclusion of Na^+^ from aerial parts and play a significant role in salt tolerance.	Xu et al. 2020
High‐affinity K^+^ transporters (HKTs)	Roots and leaves, cell membrane, tonoplast	KT/HAK/KUP	*Sorghum bicolor* (L.) *Moench*	HKTs act as cation symporters that under heterologous expression indicate slight Na^+^ permeability and salt tolerance. These transporters may exhibit high affinity for Na^+^ in plasma membrane and low affinity in tonoplast.	Guo et al. 2023
Low‐affinity Na^+^ transporters	Roots and leaves	LCT1	*Triticum aestivum*	LCT1 facilitates low‐affinity transport of Na^+^ as well as some other cations such as Rb^+^ and Ca^2+^.	Amtmann et al. (2001)
Shaker type K^+^ channels	Roots and leaves, cell membrane	AKT1 and KAT1	*Triticum aestivum*	These channels show permeability to Na^+^ in plants with increased Na^+^ concentrations.	Xue et al. (2011)
Cation‐chloride co‐transporters	Root, shoots, leaves, trichomes, anthers, cell membrane	KCC group (K^+^‐Cl^−^ cotransporter), NCC group (Na^+^‐Cl^−^ cotransporter), NKCC group (Na^+^‐K^+^‐2Cl^−^ cotransporter)	*Arabidopsis*	Cation‐chloride co‐transporters are secondary active transporters that facilitate the cotransport of at least one cation, such as Na^+^, with Cl^−^.	Colmenero‐Flores et al. (2007)
Na^+^/Ca^2+^ exchangers (NCXs)	Root, cell membrane, cell wall	AtNCL	*Arabidopsis*	Na^+^/Ca^2+^ exchangers maintain Na^+^ and Ca^2+^ homeostasis across cellular membranes by extruding excess Ca^2+^ in exchange for Na^+^, and facilitating Na⁺ uptake in plants or sequestering it into vacuoles under high salt stress conditions.	Wang et al. (2012)
Na^+^/H^+^ exchangers (intracellular) (NHXs)	Roots, leaves, stems, tonoplast, cell membrane	AtNHX1	*Arabidopsis*	Na^+^/H^+^ exchangers (NHXs) constitute one of the two families of cation‐proton antiporters that move Na^+^ via a H^+^ gradient across the cell membrane or accelerate Na^+^ compartmentalization that enhances salt tolerance.	Bassil et al. 2018
Cation/H+ exchangers (CHXs)	Roots, stems, leaves, plasma membrane and intracellular membranes	AtCHX21	*Arabidopsis*	The CHX transporter is a cation‐proton antiporter that mediates cation exchanges, including Na^+^, K^+^ and H^+^, with probably Cl^−^. They may serve multiple roles in the maintenance of pH and ionic balance including Na^+^ accumulation in leaves and adjustment of Na^+^ levels in the xylem.	Isayenkov et al. 2020
Plasma membrane‐bound Na^+^/H^+^ exchangers (NhaP)	Root, shoot, plasma membrane, tonoplast, endosomes	SOS1	*Arabidopsis*	The Na^+^/H^+^ antiporter regulates Na^+^ extrusion and long‐distance transport in plants. Moreover, overexpression of *SOS1* enhances salt tolerance.	Olías et al. 2009
Aquaporins	Roots, plasma membrane	AtPIP2;1	*Arabidopsis*	Aquaporins are proteins that have high conductance for water and are non‐selectively permeable to various cations, primarily Na^+^.	Byrt et al. (2017)
Nonselective cation channels (NSCCs)	Roots, plasma membrane	Voltage‐dependent NSCCs and voltage‐independent NSCCs	*Arabidopsis*	NSCCs mediate the passive influx of numerous cations that might lead to toxic Na^+^ influx in response to salt stress.	Demidchik and Tester 2002
Voltage independent channels (VICs)	Roots, plasma membrane	VIC	*Arabidopsis*	VIC is implicated in Na^+^ influx, exhibiting nonselective mono‐cation influx activity and voltage‐independent gating.	Maathuis and Sanders 2001
Cyclic nucleotide‐gated channels (CNGCs)	Plasma membrane	CNGC10	*Arabidopsis*	CNGCs are voltage‐independent nonselective cation channels that allow the influx of cations including Na^+^ in the presence of cyclic nucleotides (CNs).	Guo et al. 2008
Glutamate receptors (GLRs)	Plasma membrane	GLR3.7	*Arabidopsis*	Glutamate‐triggered NSCCs in plants remain poorly understood. Ionotropic glutamate receptors are potential constituents of NSCCs, capable of transporting diverse cations such as Na^+^, K⁺, and Ca²⁺.	Wang et al. 2019
Receptor‐like kinases (RLKs)	Plasma membrane	FERONIA	*Arabidopsis*	RLKs are cell wall‐associated receptors that transmit Na^+^ stress‐related signals by altering cell wall components.	Feng et al. 2018
Mechano‐sensory channels	Plasma membrane	MSL10	*Arabidopsis*	Mechanosensory channels sense changes induced in the cell in response to swelling induced by environmental stresses and lead to programmed cell death.	Basu and Haswell (2020)
Histidine kinases	Plasma membrane	AHK1, AHK2, AHK3	*Arabidopsis*	Histidine kinases play an important role in response to salt stress by regulating various salt stress‐responsive genes or abscisic acid (ABA)‐inducible genes and contribute to salt stress tolerance.	Tran et al. 2007
NADPH oxidase	Roots, plasma membrane	CsRboh	*Cucumis sativus L. var. Krak*	Salt stress exposure upregulates NADPH oxidases, leading to enhanced H_2_O_2_ production and increased activities of antioxidant enzymes.	Kabała et al. 2022

## 2 Molecularly Evident Na^+^ Sensors: Glycosyl Inositol Phosphoryl Ceramide (GIPC) Phospholipids

All plants, including glycophytes and halophytes, deploy GIPC (glycosyl inositol phosphoryl ceramide) sphingolipids as extracellular Na^+^ sensors in the plasma membrane, which trigger downstream Ca^2+^ signaling (Rahman et al. 2021). The plasma membrane (PM) comprises three main classes of lipids: sphingolipids, sterols, and phosphoglycerolipids. GIPCs are an important sphingolipid subclass constituting 40% of the PM lipids, which consists of long saturated acyl chains in the outer leaflet of the PM and form raft‐like lipid microdomains (Rahman et al. 2021).

An α‐glucuronosyltransferase, encoded via the *IPUT1* gene, synthesizes GIPC through the delivery of a glucuronic acid (GlcA) moiety from UDP–GlcA to inositol phosphorylceramide (IPC) (Rennie et al. 2014) The *iput1* mutant had low GIPC levels, causing severe dwarfism, which could be rescued with a pollen‐specific promoter‐driven *IPUT1* (Tartaglio et al. 2017). GIPCs play an important role as Na^+^ sensors. In glycophytes such as *Arabidopsis, MOCA1* encodes a glucuronosyltransferase that binds a GlcA group with a negative charge to inositol phosphorylceramide, resulting in GIPC sphingolipid formation in the plasma membrane(Jiang et al. 2019). In salt stress, GIPC binds Na^+^ and depolarizes the membrane, which triggers unknown salt‐dependent Ca^2+^ channels that generate intracellular Ca^2+^ transients upon salinity stress. This Na^+^‐GIPC‐Ca^2+^ module was proposed to be a Na^+^ sensing mechanism that triggers the downstream CBL–CIPK pathway, leading to NHX activation that promotes Na^+^ efflux. Furthermore, the absence of Ca^2+^ waves in the *moca1* mutant upon Na^+^, K^+^, or Li^+^ build‐up or salt stress affirmed this hypothesis (Jiang et al. 2019). In addition, *IbIPUT1*, an ortholog of *AtIPUT1* in sweet potato (*Ipomoea batatas*), was also found to play a positive role in salt tolerance. *IbIPUT1*‐overexpression reduced Na^+^ uptake by sweet potato roots, decreased Na^+^ accumulation, and influenced Ca^2+^ flux under salt stress (Liu et al. 2022a).

In halophytes under salt stress, such as the succulent euhalophyte *Salicornia europaea*, a significant increase in GIPC levels along with other membrane lipids was revealed in lipidomic analyses as a result of lipid remodeling (Yang et al. 2023), suggesting a possible role of GIPCs in Na^+^ sensing during salt stress. Another study on *Salicornia europaea* revealed the double‐bond index of GIPC sphingolipids to be predominantly high among membrane lipids under salt stress, compared to the control. In addition, the transcriptomic data revealed that phosphatidylinositol signaling, lipid biosynthesis, and glycerophospholipid metabolism were significantly affected under salt stress. The upregulation of genes involved in Na^+^ exclusion, i.e., *SeNHX1*, *SeSOS1*, *SeVP1*, and *SeVHA‐A*, suggests the formation of the Na^+^‐GIPC‐Ca^2+^ complex. However, this needs experimental validation (Yang et al. 2024). Although forward genetic studies of the *MOCA1* gene or GIPC biosynthesis‐related genes are still lacking in halophytes, we hypothesize that the tissue‐and organ‐specific abundances of GIPCs, their diverse glycan headgroups, and the elevated expressions of GIPC biosynthetic genes (Mamode Cassim et al. 2021) play a more important role in Na^+^ sensing in halophytes than in glycophytes. The quantitative and structural modifications of GIPCs in halophytes might enhance Na^+^ binding and the selective tolerance of halophytes (Mamode Cassim et al. 2021). However, this hypothesis needs functional and experimental validation. Nonetheless, the higher GIPC levels in halophytes than in glycophytes suggest that Na^+^ sensing by GIPCs could play a role in salt tolerance in halophytes.

## 3 Nonselective Cation Channels (NSCCs) in Na^+^ Sensing

**Figure 1:**
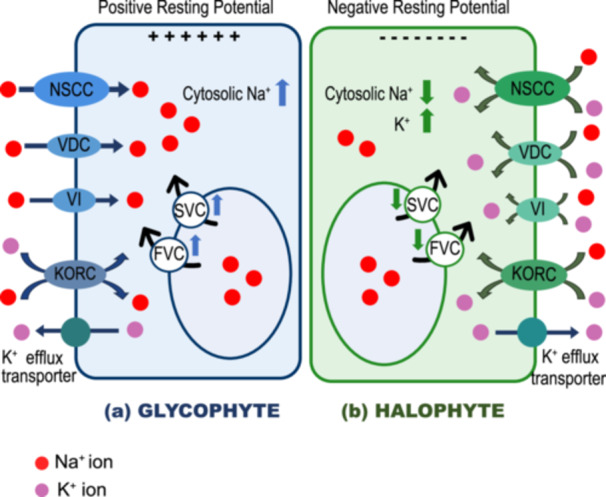
Distinctive adaptations related to sodium ion (Na^+^) transport in glycophytes versus halophytes. (a) In glycophytes, under high salt conditions, Na^+^ enters non‐selectively through various voltage‐dependent (VDC) and voltage‐independent (VI) channels, causing depolarization on the membrane and making the cell interior more positively charged. The potassium efflux transporters are activated under high salt conditions, and the cytosolic Na^+^ concentration significantly rises inside the cell. The potassium channels, such as KORC become more permeable to Na^+^ than K^+^, causing high Na^+^ influx. In addition, the slow‐voltage channels (SVC) and fast voltage channels (FVC) voltage‐dependent channel activities increase, causing significant Na^+^ efflux from vacuoles into the cytoplasm. (b) The NSCCs in halophytes possess enhanced selectivity for potassium (K^+^) over Na^+^, therefore restricting Na^+^ influx and helping to sustain ion balance in a high‐Na^+^ environment. Having a higher permeability to K^+^ when under high Na^+^ conditions maintains the negative resting membrane potential inside the cells of halophytes. The K^+^ channels in halophytes are highly impermeable to Na^+^, maintaining low cytosolic Na^+^ levels (Carpaneto et al. 2004). In addition, the activities of SVCs and VIs in the tonoplast decrease the Na^+^ efflux from the vacuole into the cytosol, significantly differentiating them from glycophytes. NSCC, nonselective cation channel; VDC, voltage‐dependent channel; VI, voltage‐independent channel; KORC, K^+^ outward rectifying conductance; SVC, slow‐activating voltage‐dependent channel; FVC, fast‐activating voltage‐dependent channel. Red circle, Na^+^; purple circle, K^+^. “↑”, increase in concentration or increase in activity; “↓”, decrease in concentration or decrease in activity. [Color figure can be viewed at wileyonlinelibrary.com]

**Figure 2:**
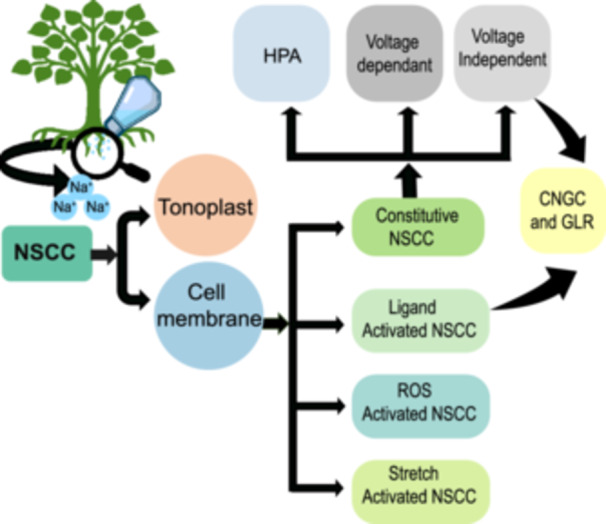
Classification of nonselective cation channels in plants related to sodium (Na^+^) sensing. Na^+^ ions diffuse into the root cells of plants through nonselective cation channels (NSCC). NSCCs are found in the tonoplast as well as the plasma membrane. The NSCCs in the plasma membrane include constitutive NSCCs, reactive oxygen species (ROS)‐activated channels, ligand‐activated channels that include cyclic nucleotide cation channels (CNGCs) and glutamate receptors (GLRs), and mechanosensitive or stretch‐activated channels. The constitutive NSCCs involved in Na^+^ influx can be categorized as hyperpolarization‐activated (HPA) channels, depolarization, that is, voltage‐dependent, channels that cause the cell interior to become more negatively charged, and voltage‐independent channels that allow Na^+^ flux regardless of the voltage difference across the cell membrane. [Color figure can be viewed at wileyonlinelibrary.com]

Halophytes and glycophytes possess fundamentally different plasma membrane NSCC‐mediated Na^+^ sensing strategies. NSCCs, as the name suggests, are a heterogeneous group of macromolecular pores that exhibit very low or minimal selectivity for essential (Na^+^, K^+^ and Ca^2+^) as well as toxic cations (Demidchik et al. 2002), with rates exceeding 10^7^ cations per channel pore per second. NSCCs are also present in tonoplast, mitochondria, chloroplasts, endomembrane, and symbiosomal membranes (Lee et al. 2018). NSCCs in halophytes and glycophytes exhibit distinct characteristics, as shown in Figure 1. NSCCs cannot be defined by a single criterion. In this review, NSCCs were classified according to their Na^+^ selectivity, mainly focusing on cyclic nucleotide‐gated channels (CNGCs) and glutamate receptors (GLRs), particularly those in plasma membranes. A schematic of this classification is shown in Figure 2.

NSCCs are the principal Na^+^ sensors that mediate passive Na^+^ influx into roots. However, halophytes modulate NSCC‐mediated Na^+^ entry differently than glycophytes. Halophytes tightly regulate NSCCs under salt stress, which either reduce net Na^+^ uptake via roots, or limit Na^+^ root‐to‐shoot translocation, or affect Na^+^ cellular partitioning (Maathuis 2013). The comparative electrophysiological studies indicate reduced Na^+^ influx and higher K^+^/Na^+^ selectivity of NSCCs in halophytes as compared to glycophytes, which contributes to halophytes' superior tolerance to salt stress (Volkov et al. 2004; Flowers and Colmer 2008). However, high variability exists in the NSCC regulation during excess Na^+^, depending on plant species, tissue specificity, or other physiological factors in halophytes (Volkov 2015). In contrast, glycophytes exhibit minimal NSCC regulation that results in cytotoxic Na^+^ accumulation. For instance, in *Arabidopsis*, NSCCs mediate Na^+^ influx at the rate of 30 nmol·g^−^¹ FWs^−^¹ at 100 mM, which leads to rapid cytosolic Na^+^ buildup and causes cytotoxicity due to Na^+^ accumulation (Volkov et al. 2004). However, a halophytic relative of *Arabidopsis*, *T. halophila* (salt cress; a salt excluder), showed less than half of the unidirectional Na^+^ influx rates than those of *Arabidopsis*. *T. halophila* NSCCs managed to reduce passive Na^+^ entry, minimizing the need for costly Na^+^ extrusion or sequestration (Volkov et al. 2004).

Halophytes possess modified NSCC gating properties and expression patterns different from glycophytes. For instance, voltage‐independent NSCC currents in *T. halophila* showed higher selectivity for K^+^ over Na^+^ and better stomatal control (lower stomatal conductance) under saline conditions than those in *Arabidopsis*, suggesting different channel composition or regulatory subunits (Volkov and Amtmann 2006). In addition, *Zostera marina L*. (eelgrass; a salt excluder) possesses NSCCs with Na^+^/K^+^ selectivity in leaf cells under high Na^+^ conditions. Electrophysiological analyses revealed lower permeability for Na^+^ than for K^+^ by these NSCCs, allowing a negative resting potential inside cells even under high Na^+^ conditions (Fernandez et al. 1999). The ion channels in the plasma membrane of the roots of *T. halophila* were found to have high K^+^/Na^+^ selectivity and restricted Na^+^ uptake in halophytes in high‐salt environments (Volkov and Amtmann 2006). Moreover, these ion channels also facilitate K^+^ uptake and are inhibited in the presence of external Ca^2+^ under salinity stress (Volkov et al. 2004). In contrast, salt‐tolerant and salt‐sensitive *Plantago sp.(Plantago media L., P. coronopus L)* (fleawort; a salt accumulator) showed similar rates of Na^+^ uptake, suggesting that the survival of salt‐tolerant species can be due to the translocation of Na^+^ from roots to shoots by NSCCs to prevent the overloading of Na^+^ sequestered in vacuoles (Erdei and Kuiper 1979).

A comparison of NaCl‐induced programmed cell death (PCD) between the cell cultures of *Cakile maritima* (Sea Rocket); an obligate halophyte and a salt excluder) and *Arabidopsis thaliana* showed more efficient Na^+^ regulation through NSCCs in *C. maritima*. The NSCCs in *C. maritima* behaved in one of two distinct ways: either led to a sustained depolarization of *C. maritima* cells that resulted in caspase‐like‐dependent PCD or to transient depolarization, causing a gradual reduction in NSCC activities that improved cell survival under salt stress. In addition, *C. maritima* cells had more efficient NSCC‐dependent Na^+^ management than *A. thaliana* (Ben Hamed‐Laouti et al. 2016). It was suggested that a second pathway may involve an Na^+^ exclusion system, such as SOS, that aids in plant survival (Arbelet‐Bonnin et al. 2018). A comparative study on the guard cells of the halophyte, *Aster tripolium* (Sea Aster); a salt accumulator. and its non‐halophytic relative, *Aster amellus* (Italian Aster), showed a weakly instantaneous Na^+^‐permeable cation channel that accumulates cytosolic Na^+^. This increased Na^+^ accumulation inside the guard cells, causing stomatal opening in the non‐halotolerant plant *A. amellus*, resulting in detrimental effects. In contrast, the halophyte, *A. tripolium*, had a low level of cytosolic Na^+^ accumulation and exhibited only partial stomatal opening (Véry et al. 1998). The study proposed that this Na^+^‐induced differential stomatal regulation might be attained by the specific mechanism of Na^+^ sensing in *A. tripolium* that is absent *in A. amellus*, followed by a signal transduction pathway that alleviated cytosolic Ca^2+^, which then inactivated K^+^ channel activities and enhanced the salt tolerance of the halophyte (Véry et al. 1998). A comparison between the salt‐tolerant *Plantago maritima* (sea plantain)(salt accumulator) and the salt‐sensitive *Plantago media* (hoary plantain) showed different magnitudes of membrane depolarization caused by Na^+^ and K^+^ between the two species, with lower permeability in the salt‐tolerant plant (Maathuis and Prins 1990).

The uptake of Na^+^ during salt stress is followed by the downstream active exclusion (via SOS1‐type Na^+^/H^+^ antiporters) or ion sequestration into vacuoles, both processes that incur ATP or proton motive force expenditure, imposing growth penalties on plants in general (Ramakrishna et al. 2025). For instance, in *Arabidopsis* root meristem cells, SOS1(/NHX7) mediates the extrusion of Na^+^ ions at lower salt concentrations. However, at higher NaCl (100 mM) concentration, Na^+^ toxicity is prevented via Na^+^ sequestration in the vacuoles rather than Na^+^ extrusion via the SOS1 protein in both meristem and differentiated cells. In addition, in rice, vacuolar sequestration of Na is conserved in root meristem cells via a vacuolar‐localized SOS1 homolog. The OsSOS1 mediates the vacuolar Na sequestration in differentiated root tissue (Ramakrishna et al. 2025).

Certain halophytes, namely recretohalophytes, in addition to the above mechanisms, can dump Na^+^ externally on the leaf surface via specialized epidermal bladder cells (e.g., *Mesembryanthemum crystallinum;* common ice plant and a sodium accumulator) or via salt glands that glycophytes lack (Oh et al. 2015). In addition, certain halophytes, for instance, the euhalophyte *Salicornia bigelovii* (common glasswort), can neolocalize the SOS1‐NHX homolog (SbiSOS1) into the tonoplast from the plasma membrane to direct vacuolar loading and prevent cytotoxicity (Salazar et al. 2024). Glycophytes expend more energy on Na^+^ exclusion and yet are unable to achieve the low rates of net Na^+^ uptake as in halophytes. One reason can be the ability of halophytes to restrict Na^+^ uptake via nonselective cation channels (NSCCs), thus reducing the need for the subsequent energy‐demanding Na^+^ efflux from roots, as evidenced in a comparative study of *Arabidopsis* and *T. halophila* (Volkov et al. 2004). Furthermore, *T. halophila* has a more negative membrane potential (−80 mV) than *Arabidopsis* (−40 mV) upon salt stress, implying it has less energy expenditure in maintaining a negative membrane potential under sodic stress as compared to the energy expended by glycophytes (Volkov and Amtmann 2006). In addition, the salt‐accumulating halophyte *Salicornia dolichostachya* (Long‐spiked Glasswort) has constitutively higher levels of SOS1 in roots as well as tonoplast NHX1 and VHA‐subunit C in leaves, compared to these levels in the glycophyte *Spinacia oleracea* (spinach). Under excess Na^+^, SdSOS1 functions in the xylem loading of Na^+^, increasing the Na^+^ level in shoots and its subsequent accumulation in leaf vacuoles. The constitutively high levels of tonoplast NHX1 and VHA‐subunit C reduce the energy expenditure via ATP hydrolysis for maintaining the electrochemical H^+^ gradient across the plasma membrane, thus allowing the accumulation of Na^+^ in vacuoles without growth penalties (Katschnig et al. 2015).

NSCCs in halophytes possess a unique Na^+^ sensing signature that distinguishes them from glycophytes via restrictive Na^+^ entry and high K^+^/Na^+^ selectivity under high salt conditions. It is important to identify cis‐regulatory elements and post‐translational modifications, as well as structural differences that confer variations among different halophyte classes (whether salt excluders or salt accumulators) and from glycophytes.

## 4 CNGCs as Potential Na^+^ Sensors

**Figure 3:**
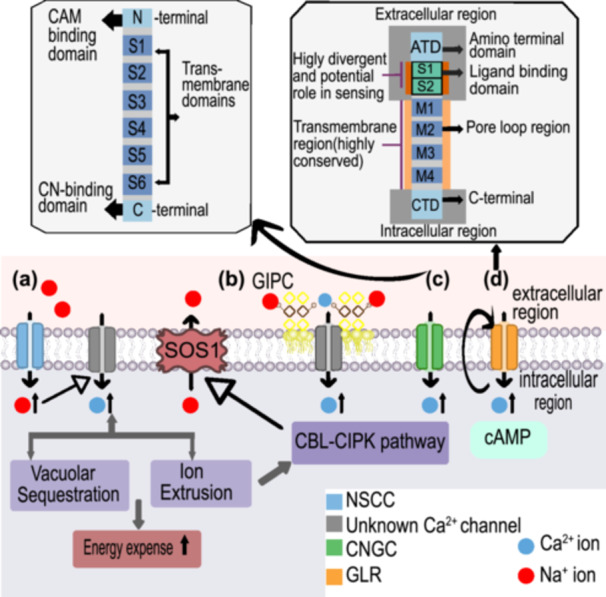
General overview of sodium (Na^+^) sensing mechanisms in plants. (a) Na^+^ entry through nonselective cation channels (NSCCs) leads to downstream Ca^2+^ signaling and Na^+^ management via sequestration or extrusion. (b) Direct binding of Na^+^ to glycosyl inositol phosphoryl ceramides (GIPCs) in the cell membrane initiates Ca^2+^ signaling. (c) General structure of cyclic nucleotide‐gated channel (CNGC), potentially involved in Na^+^ sensing and inducing Ca^2+^ signaling. Their structure contains an N‐terminal CaM‐binding domain followed by six conserved transmembrane domains (designated S1–S6), a hydrophilic C‐terminal CN‐binding domain, and, in some cases, an ankyrin domain (d). General structure of glutamate receptor (GLR) potentially participating in sensing Na^+^ and mediating Ca^2+^ signaling. The M1, M2 and M3 regions of the transmembrane domain are highly conserved among all four clades, while the extracellular LBD (Ligand binding domain) is highly divergent among plants. The transmembrane domain of a GLR contains pores for ion channels and is an important structure for ion selectivity. The M2 region is the most critical for GLR functions, particularly in determining ion selectivity. The amino acid sequences in the ATD (Amino terminal domain) and LBD are extremely divergent; however, their modes of function are surprisingly similar, suggesting that these extracellular signals are sensed through the LBD and ATD of GLRs. [Color figure can be viewed at wileyonlinelibrary.com]

Both halophytes and glycophytes mediate Na^+^ flux via CNGCs (cyclic nucleotide‐gated channels), inducing Ca^2+^ signaling. However, halophytes might exhibit either structural or regulatory modifications that limit Na^+^ conductance, acting as important Na^+^ sensors (Assaha et al. 2017). CNGCs are nonselective ion channels that are activated upon CN (cAMP or cGMP) binding (Demidchik and Maathuis 2007). They have structural similarities with the Shaker‐type channels and possess common binding sites for CaM (Calmodulin) and CNs (cyclic nucleotides), facilitating the crosstalk between CaM and CN signaling (K. Jha et al. 2016) as shown in Figure 3.

Information on the molecular or electrophysiological investigations of CNGCs in halophytes is limited as compared to glycophytes (Shabala and Mackay 2011). Therefore, our understanding of CNGCs in halophytes primarily relies on the transcriptomic studies of these plants. CNGCs might also have a decisive role in sensing Na^+^ in halophytes, just like in glycophytes, given their roles in ion homeostasis regulation (Assaha et al. 2017), cation (Na^+^ and K^+^) uptake and distribution in saline conditions, differential gene expressions of Na^+^ and K^+^ channels and transporters (Ma et al. 2010). Moreover, CNGCs mediate cellular stress responses to homeostasis, which implies that they might play a role in aiding halophytes to sense and respond to Na^+^ stress (Jarratt‐Barnham et al. 2021). The transcriptomic data suggest high variability in CNGCs in halophytes as well as glycophytes. Distinct isoforms of CNGCs exist in halophytes that are either upregulated or downregulated upon ionic stress exposure, indicating more robust induction of CNGCs in halophytes as compared to glycophytes (Gharat et al. 2016). A list of putative CNGC‐type Na^+^ sensors in other plant species is provided in Table S1.

Several electrophysiological studies in glycophytes indicate the role of CNGCs in Na^+^ perception. For instance, a patch clamp inside‐out experiment on *Xenopus laevis* oocytes to study the gene product of *HLM1/CNGC4* showed that the cGMP‐ and cAMP‐activated CNGC4 ion channel was permeable to both K^+^ and Na^+^, suggesting its possible role in sensing Na^+^ (Balagué et al. 2003). CNGC1 and CNGC10 participate in the transport of cations and heavy metals in *Arabidopsis* (Gobert et al. 2006). Even though their physiological characteristics remain unclear, the presence of mono‐cation activity accounts for the possibility of Na^+^ conductance by these channels (Gobert et al. 2006). *AtCNGC1* and *AtCNGC2* from *Arabidopsis* and *NtCBP4* from tobacco were cloned into a heterologous expression system in *Xenopus* oocytes or human HEK 293 cells, and the electrophysiological analyses of these transgenic cells demonstrated K^+^ selectivity, with *AtCNGC2* conducting K^+^ and other monovalent cations while specifically excluding Na^+^ (Leng et al. 2002). CNGC10 is permeable to Na^+^ and K^+^. The *cngc10* mutant had reduced accumulation of Na^+^ in shoots. Moreover, GUS analyses of the root cross‐section of *Arabidopsis* showed *CNGC10* expression in the root epidermis and endodermis. The yeast mutant strain B31 overexpressing *CNGC10* from *Arabidopsis* exhibited Na^+^ sensitivity and showed a reduction in growth due to a higher rate of Na^+^ accumulation than yeast transformed with an empty vector. Furthermore, the exposure of *Arabidopsis* roots to 200 mM NaCl for 6 h significantly repressed *CNGC10* expression. These findings revealed *CNGC10* to be a negative regulator of salt tolerance in *A. thaliana* and implicated it in sensing Na^+^ (Jin et al. 2015). In addition, *CNGC3*, GUS staining demonstrated its predominant expression in the epidermal and cortical root tissues of seedlings, serving as a likely pathway for the uptake of both Na^+^ and K^+^ in heterologous systems, where a loss‐of‐function mutation of *CNGC3* resulted in the diminished uptake of Na^+^ in root tissue of *Arabidopsis* seedling (with a significant but temporary loss) upon exposure to 40–80 mM NaCl (Gobert et al. 2006). *CNGC19* and *CNGC20* modulated cation fluxes in plants during salt and biotic stresses. However, more experiments to elucidate their roles in Na^+^ sensing are desired to *disentangle the direct roles of CNGC19 and CNGC20 in the subcellular redistribution of Na* ^*+*^ (Yuen and Christopher 2013). These findings suggest the involvement of CNGCs in the perception of Na^+^ in glycophytes.

Several studies in halophytes indicate that CNGC is involved in Na^+^ perception. For instance, a comparative analysis revealed that *Zygophyllum xanthoxylum* (caper beans), a salt accumulator, contains nine CNGC1 orthologs (ZxCNGC1s), in contrast to a single CNGC1 *Arabidopsis* (AtCNGC1) ortholog, despite containing similar motifs. The expression of *ZxCNGC1;1* and *ZxCNGC1;2*, in yeast strain G19, followed by a patch clamp experiment in HEK 293T cells, showed higher Na^+^ permeability as compared to *OsCNGC1* (Ma et al. 2024a). In contrast, AtCNGC1 in *Arabidopsis* had no visible Na^+^ current. ZxCNGC1 mediates Na^+^ uptake through a unique energy‐saving pathway that does not require the activity of H^+^‐ATPases to provide energy to maintain the hyperpolarization‐dependent membrane potential, while glycophytes and some other halophytes need the energy supplied by H^+^‐ATPases for the hyperpolarization‐dependent uptake of Na^+^ (Ma et al. 2024a).

The comparative study between *Arabidopsis*, a glycophyte, and *T. halophila*, a salt‐excluding halophyte, identified *CNGC8* as a potential candidate for controlling Na^+^ influx. Transcriptional analyses through DNA microarray revealed the differential downregulation of *CNGC8* between these two species under salt stress (Wang 2006). In *T. halophila*, *CNGC8* was downregulated in roots, while *CNGC8* in *Arabidopsis* was downregulated in shoots. This suggests that *T. halophila* might reduce Na^+^ toxicity by minimizing Na^+^ influx through roots, which is consistent with the low Na^+^ accumulation in the roots of *T. halophila* under salt stress. In contrast, *Arabidopsis* triggers various transcriptional responses to reduce Na^+^‐induced toxicity in response to high apoplastic Na^+^ in shoots. Nevertheless, further functional characterization of *CNGC8* is needed to confirm its role in Na^+^ uptake (Wang 2006). *CNGC14*, which facilitates the absorption and subsequent excretion of Na^+^ to inhibit salt‐induced damage, was significantly upregulated in the salt bladder of *Atriplex centralasiatica* (Central Asian Saltbush; a salt excluder) (Yao et al. 2019).

Five CNGCs were upregulated in *Limonium bicolor* (bicolored sea lavender; a salt excretor) and participated in the Na^+^ excretory pathway by increasing Na^+^ uptake from the surrounding apoplastic region into the cytosol of salt glands in the leaf epidermis. Inside the cytosol, the Na^+^ ions were transported into vesicles via either NHXs or through Na^+^–K^+^–Cl^−^ co‐transporters, and then extruded to the leaf surface by vesicle fusion with the plasma membrane (Yuan et al. 2016). A transcriptomic study of *Pugionium cornutum* (horned Pugionium), a salt‐accumulating halophyte, revealed differential expressions of several *CNGC*s in roots and shoots under salt stress after 6 and 24 h. CNGCs regulate ion homeostasis and are permeable of Na^+^, Ca^2+^, and K^+^ ions (Cui et al. 2019). Significant salt‐induced upregulation of *CNGC4* and downregulation of two *CNGC17* genes in *Suaeda dendroides* (tree seablite or treelike seablite; salt excluder), a native halophyte in China, were also uncovered (Ma et al. 2024a).

In contrast to glycophytes, certain CNGCs (e.g., CNGC8 in *T. halophila*) in halophytes showed significantly diverse responses to salt, with predominant downregulation upon salt exposure (Wang 2006). Their varying transcriptional responses suggest that their regulation depends on the plant species and the stage of the salt stress. This feature might account for their survival in extremely saline environments. Deciphering the intricacies in these regulatory mechanisms may contribute to enhancing salt tolerance in glycophytes. In addition, the transcriptomic distinctions suggest that CNGCs in halophytes have evolved both quantitative (higher or lower expression levels) and qualitative (expanded paralog diversity, altered tissue‐specific regulation) traits that mediate Ca^2+^‐induced Na^+^ sensing (Yong et al. 2014). Expression of halophytic *CNGC* genes in glycophytic crops will require well‐balanced *CNGC* induction with energy cost control to prevent growth penalties from Ca^2+^ cycling and downstream ion pumping demands (Assaha et al. 2017).

## 5 Glutamate Receptors (GLRs) as Potential Na^+^ Sensors

GLRs in halophytes and glycophytes mediate Ca^2+^‐dependent Na^+^ influx and act as potential Na^+^ sensors. *GLR* genes in halophytes exhibit differential expression upon salt stress. GLRs are ionotropic nonselective receptors, activated through ligand binding, and have eminent homology with the GLRs abundant in the nervous systems of animals (Simon et al. 2023). A comprehensive and absolute definition of GLRs in plants is subtle and still needs to be established (Simon et al. 2023). GLRs are localized in the plasma membrane, the endomembrane, the plastid, the endoplasmic reticulum, and the tonoplast (Alfieri et al. 2020). While the basic structure of GLRs is conserved, it is hypothesized that halophytes may possess specific structural modifications that efficiently regulate Na^+^ fluxes, given the multiple homologs in halophytes. (Alfieri et al. 2020). These structural changes might reflect alterations in channel selectivity and gating. However, the specific structural differences in GLRs between halophytes and glycophytes have to be elucidated.

Electrophysiological evidence indicates the potential role of GLRs as Na^+^ sensors. In the model plant *A. thaliana*, 20 GLRs have been characterized. The overexpression of *AtGluR2* (also known as *AtGLR3.2*) in transgenic *Arabidopsis*, driven by a 35S promoter, resulted in impaired Ca^2+^ signaling and showed evidence of hypersensitivity to ionic stresses, including Na^+^ and K^+^. Moreover, this hypersensitivity was restored by the augmentation of Ca^2+^ (Kim et al. 2001). The antisense‐*AtGLR1.1 Arabidopsis* plants exhibited decreased hypersensitivity to Na^+^ and K^+^ compared to wild‐type plants (Kang and Turano 2003). The addition of glutamate accelerated the unidirectional influx of Na^+^ through glutamate‐activated NSCCs in the intact roots of *Arabidopsis*, and the cells activated by glutamate had larger Na^+^ currents (Demidchik et al. 2004). A membrane transplantation experiment to determine the ion conductivity of the pore domains of *Arabidopsis* GLR (AtGLR) in the *Xenopus* oocyte heterologous expression system in the presence of glutamate found that the pore domains of AtGLR1.1 and AtGLR1.4 have permeability to Na^+^, K^+^ and Ca^2+^, verifying their nonselective ion activities (Tapken and Hollmann 2008).

The *AtGLR3.4*‐homolog knockout mutants, *Atglr3.4‐1* and *Atglr3.4‐2*, showed greater sensitivity to NaCl through seed germination and subsequent growth stages. These mutants had compromised salt‐induced increases in cytosolic Ca^2+^, verifying that AtGLR3.4 facilitates the accumulation of Na^+^ in seeds during germination under salt stress (Cheng et al. 2018). A list of GLRs that have potential Na^+^ sensing activities in other plant species than *Arabidopsis* is provided in Table S2. More in‐depth investigations are needed to determine how GLRs in halophytes perceive Na^+^ during salt stress.

Several transcriptomic studies in halophytes indicate differential *GLR* expression under salt stress. A differential gene expression (DEG) analysis of *Nitraria sibirica* Pall. (Nitre Bush; a euhalophyte) under 300 mM salt stress, indicated the differential expression of four *GLR* paralogs. Among these, three *GLR*s were downregulated while one *GLR* was upregulated, decreasing Na^+^ inflow under salt stress and reducing Na^+^‐induced damage (Zhang et al. 2023). The genomic analysis of *Tamarix chinensis* (Chinese tamarisk and five‐stamen tamarisk), a recretohalophyte, under 300 mM NaCl stress revealed expanded gene families involved in salt stress sensing and ion homeostasis. In particular, a whole‐genome duplication of the genes encoding *GLR 3.2* implicates its role in Na^+^ sensing (Liu et al. 2022c). A study on the xerophyte *Pugionium cornutum* (horned Pugionium; a salt accumulator) demonstrated that GLR was upregulated under salt stress and had a role in the uptake of Na^+^, Ca^2+,^ and K^+^ in roots. In addition, GLR3.3 was particularly upregulated after 6 and 24 h of salt treatment in shoots (Cui et al. 2019). The transcriptomic analyses of *Chenopodium quinoa* (goosefoot), a salt accumulator, indicated that *GLR2.8* and two homologs of *GLR 2.7* were significantly upregulated (by 3.37–42.1 folds) in leaves containing epidermal bladder cells (EBC) in comparison to quinoa leaves without EBC under 400 mM NaCl stress (Kiani‐Pouya et al. 2022). In addition, RNA‐seq of a salt‐accumulating halophyte, *Sesuvium portulacastrum* (sea purslane), identified nine differentially expressed *GLR*s under 400 mM salt stress (Wang et al. 2022b). Direct functional assays and forward or reverse genetic studies, and electrophysiological studies indicating the structural modifications should be conducted. It is important to determine halophyte GLR ligand selectivity and gating properties, as it will help elucidate whether GLRs function in avoiding excessive Ca²^+^ spikes at high Na^+^ concentrations or by interacting with any downstream regulatory proteins to fine‐tune their responses to excessive Na^+^ (Kiani‐Pouya et al. 2022).

## 6 Receptor‐Like Kinases (RLKs) as Potential Na^+^ Sensors

Plants, including halophytes and glycophytes, contain RLKs that perceive Na^+^ and induce downstream signaling. The halophytes, however, exhibit expansions in lineage‐specific RLK subfamilies, multiple paralogs, gene duplications, posttranscriptional/posttranslational modifications, variations in protein abundances, and gene expression patterns that impact downstream signaling, including ion homeostasis, ROS homeostasis and scavenging, transcription reprogramming, as well as secondary metabolites and energy metabolisms (Bazihizina et al. 2021). RLKs have significant structural similarities to receptor tyrosine kinases found in animals, due to similarities in structural domains. A comprehensive study of the kinome (a collection of protein kinases encoded in the genome) that responds to salt stress is essential for understanding the sensing and perception of ions in plants (Nishiguchi et al. 2002).

Based on the diverse extracellular domains and motif arrangements in the extracellular ligand binding domain (ELCB) region, RLKs are classified into 14 different subfamilies. All members of a subfamily sense a similar type of environmental cues (Nissen et al. 2016). Among the subfamilies, leucine‐rich repeat extensins (LRXs), *Catharanthus roseus* RLK1‐like kinases (CrRLK1Ls), cysteine‐rich repeat domain‐containing receptor‐like kinases (CRKs), lysine motif receptor‐like kinases (LysM‐RLKs), wall‐associated kinases (WAKs), lectin domain‐containing receptor‐like kinases (LecRLKs), and leucine‐rich repeat‐receptor‐like kinases (LRR‐RLKs) participate in perception and tolerance to salt stress (Gandhi and Oelmüller 2023).

**Figure 4:**
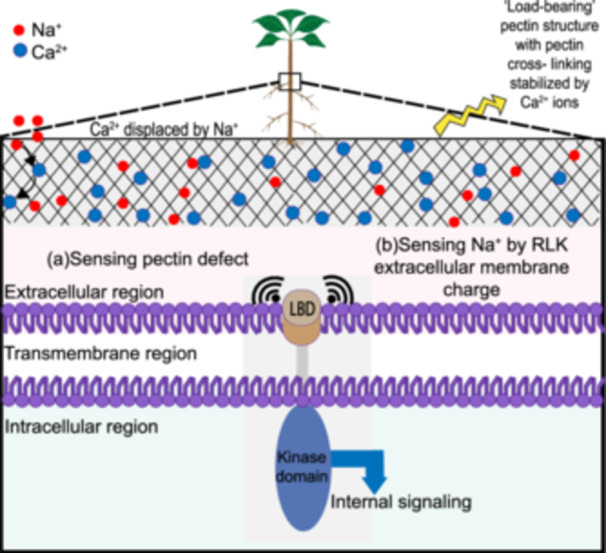
Two possible mechanisms of sodium (Na^+^) sensing through receptor‐like kinases (RLKs) in the plasma membrane. (a) Excess Na^+^ during salt stress might reduce the stiffness of the cell wall structure by modifying the cellulose microfibrils, interlinked by hemicellulose and pectin. Excess Na^+^ disrupts the pectin structure by preventing the crosslinking of polygalacturonic acid (PGA) with divalent calcium ions (Ca^2+^) and destroying the “egg carton model” of the pectin structure that supports the load‐bearing capacity of the cell wall (Feng et al. 2016; Höfte et al. 2012). These defects in cell wall structure might be sensed by the extracellular domain of RLKs present in the cell membrane or pectin‐interacting RLKs and induce downstream signaling in response to cell wall softening (Feng et al. 2018) (b) The extracellular domains of RLKs in the plasma membrane are either positively or negatively charged. The negatively charged extracellular domains might bind directly to positively charged Na^+^ and initiate downstream signaling. [Color figure can be viewed at wileyonlinelibrary.com]

RLKs do not directly perceive the abiotic stress, such as ionic, drought or temperature stress, but instead they perceive the secondary signals produced by these stresses and respond to them accordingly (Liang and Zhou 2018). After perceiving extracellular Na^+^, these signaling cascades usually result in an increase in Ca^2+^ and phytohormones, which then regulate and restore ion homeostasis, osmotic balance, and activate stress‐responsive gene expressions (Osakabe et al. 2013). Since RLKs sense Na^+^ stress indirectly, we hypothesize that there might be two ways by which RLKs perceive Na^+^ stress. They may sense Na^+^ via direct extracellular binding based on the charge of the extracellular domain, or they may perceive changes in the cell wall pectin induced by Na^+^ fluxes or stress and transduce these signals inside the cell through cell wall modifications and the signaling cascade (Figure 4).

The ELCB domain of an RLK might detect Cl^–^ when it carries a positive overall charge, whereas it can sense Na^+^ when it carries a net negative charge (Wang et al. 2022a; Isayenkov and Maathuis 2019). The ELCB domains of RLKs are located in the plasma membrane, and the extracellular domains of most RLKs are negatively charged. However, this hypothesis needs further evaluation as there is insufficient evidence to demonstrate a definite binding of Na^+^ by the extracellular domains of RLKs (Wang et al. 2022a). Therefore, to discover the potential Na^+^‐sensing RLKs, researchers mainly rely on the differential expressions of the relevant RLKs in response to NaCl stress, or the relative ion leakage or change in the relevant ion concentrations upon changing the expressions (by upregulating or downregulating) of *RLK* genes in the presence of excess salt in the apoplast (Gandhi and Oelmüller 2023).

In addition, Na^+^ reduces cell wall rigidity by perturbing ionic interactions in the cell wall and changing the pectin composition that modifies the crosslinking of cellulose in the cell wall matrix (Feng et al. 2016). An excess of the smaller monovalent Na^+^ likely replaces the larger divalent Ca^2+^ needed for the integrity of the pectin matrix and disrupts the pectin cross‐linking between adjacent carboxyl residues (Feng et al. 2016). This results in the loss of pectin molecules from the cell wall cellulose matrix, and consequently reduces the xylose/rhamnose fraction, which modulates the mechanical as well as the chemical interactions of the cell wall with the external environment, as well as initiating the perception of stress signals to trigger the early signaling cascades (Alabdallah et al. 2024). Table S3 lists all the available studies on these RLKs and the effects of salt stress in various plant species. In particular, RLKs with carbohydrate‐binding domains, found in various RLK subfamilies, may play a crucial role in transmitting the information about cell wall deformation to the cell interior via the kinase‐dependent phosphorylation of their target proteins (Gish and Clark 2011).

Feronia (FER) is a member of the CrRLK1L subfamily of RLKs, with a malectin domain that can potentially sense Na^+^ via interacting with the pectin structure in the cell wall. Na^+^ leads to the softening of the cell wall structure by disrupting the dimerization of a type of pectin, RG‐II (rhamnogalacturonan II), while FER restores the cell wall composition after exposure to salt stress. FER might interact with polysaccharides in the cell wall and sense Na^+^‐induced changes to initiate the Ca^2+^ wave necessary for cell survival (Feng et al. 2018). The binding of FER and pectin in vitro suggests that FER might physically interact with pectin in the cell wall. FER interacts with LLG1 (LORELEI‐like glycosylphosphatidylinositol‐anchored protein 1), which mediates the localization of FER to the plasma membrane and induces a salt tolerance pathway distinct from the SOS (Salt Overly Sensitive) pathway. Na^+^ might directly affect the FER‐pectin interaction or interrupt the interaction of FER with other signaling components such as the RALF peptides (Feng et al. 2018). The FER signaling model for recovery from salt stress was expanded to involve pectin, RALF1, and LLG1 (Liu et al. 2024). Salt stress triggers the formation of RALF1‐pectin phase separation in the apoplast, which acts as a sensor and forms molecular condensates. This induces the clustering of FER and LLG1 on the interface between the cell wall and cell membrane, forming nanodomains and triggering the endocytosis of FER and LLG1, which in turn triggers downstream responses for cell survival under salt stress (Liu et al. 2024). Salt stress triggers PME activation, causing the de‐methylesterification of pectin, which is detected by the cell wall sensors FER, Hercules receptor kinase 1 (HERK1), and Thesus1 (THE1), which are negative regulators of salt stress‐responsive genes that are induced via the mitogen‐activated protein kinase 6 (MAPK6) activation pathway. A model in which FER or a combination of HERK1 and THE1 attenuates the salt‐induced responses in the cell was proposed, but the roles of HERK1 and THE1 in this pathway are obscure (Gigli‐Bisceglia et al. 2022).

LRXs are a group of cell‐wall proteins consisting of an N‐terminal leucine‐rich repeat (LRR) domain and a C‐terminal extensin domain. They are positioned only in the extracellular region and possess no intracellular domain (Borassi et al. 2016). The LRR domain probably binds ligands, while the extensin domain crosslinks the components of the cell wall, such as charged pectins (Borassi et al. 2016). However, under salt stress, RALF22/23 dissociates from LRX3/4/5 via SITE‐1 PROTEASE (S1P) that cleaves the RRXL motif and releases the mature RALF22 peptides that cause endosomal FER internalization to regulate growth and salt stress responses (Zhao et al. 2018), by lowering the levels of phytohormones such as abscisic acid (ABA), jasmonic acid (JA), and salicylic acid (SA) and reducing ROS accumulation, thus playing an important role in regulating the expression of stress‐responsive genes (Zhao et al. 2018).

LRR‐RLK sub‐family that senses Na^+^‐induced damage in the cell wall resulting from the inhibition of cellulose synthesis, and contributes to salt tolerance by modulating stress‐responsive genes, lignin deposition in the cell wall, and JA accumulation (Van der Does et al. 2017). RLK7 is an LRR‐RLK that senses Na^+^ stress and interacts with PIP3 generated by salt stress to form the PIP3‐RLK7 complex, which accumulates in the plasma membrane to maintain ion homeostasis in the cell via the activation of mitogen‐activated protein kinases 3 and 6 (MPK3 and MPK6), contributing to salt tolerance (Zhou et al. 2022). Upon sensing Na^+^, CERK1 (chitin elicitor receptor kinase 1) interacts with annexin1 (ANN1) in the plasma membrane, which acts as a Ca^2+^ channel to produce a Ca^2+^ signal that upregulates downstream salt‐responsive genes (Espinoza et al. 2017).

LecRLKs may also be Na^+^ sensors. An example is *AtLecRLK2* from *Arabidopsis*, which is highly induced by salt stress and ethylene and may be involved in the transduction of extracellular Na^+^ signals to intracellular ones through its serine/threonine kinase activity in an ethylene‐dependent manner (He et al. 2004). A model was proposed based on the Na^+^‐specific response to salt stress in roots and shoots by *PsLecRLK* in *Pisum sativum*, in which the lectin domain is hypothesized to perceive various extracellular signals, such as plant hormones or oligosaccharides, and transmit these signals inside the cell to activate downstream pathways that regulate salt stress through the phosphorylation or de‐phosphorylation of proteins. However, the actual detailed pathway *in planta* is yet to be discovered (Joshi et al. 2010). Another example is OsLecRLK from rice, which positively regulates salt stress by sensing and excluding Na^+^, reducing cytosolic Ca^2+^, ROS accumulation, and lipid peroxidation, upregulating the ROS scavenging enzymes, APX (l‐ascorbate peroxidase) and SOD (superoxide dismutase), lowering catalase (CAT) activities, and accumulating osmolytes under salt stress conditions (Passricha et al. 2020).

**Figure 5:**
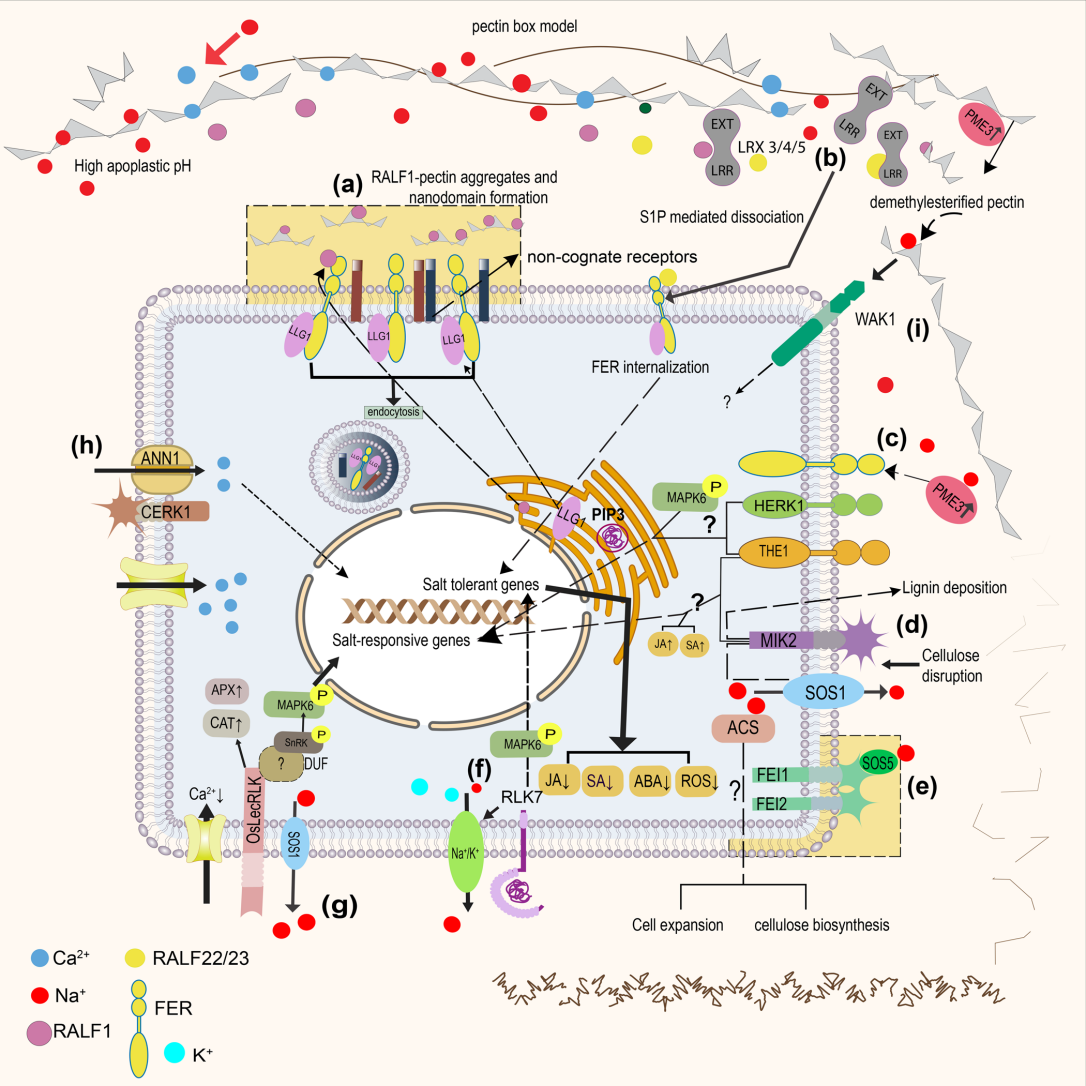
A schematic model representing the roles of receptor‐like kinases (RLKs) in sensing sodium (Na^+^) stress and the downstream responses. (a) Pectin‐RALF1‐FER‐LLG1 module; (b) RALF22/23‐LRX3/4/5‐FER internalization; (c) FER‐HERK1‐THE1 MAPK complex; (d) cellulose biosynthesis inhibition‐MIK2‐SOS1 module; (e) SOS5‐FEI1/FEI2‐ACS‐cellulose biosynthesis module; (f) PIP3‐RLK7‐MAPK‐Na^+^/K^+^ complex; (g) OsLecRLK‐SnRK1‐MAPK‐SOS1; (h) CERK‐ANN1‐Ca^2+^‐induced gene activation; (i) Na^+^‐pectin‐PME‐WAK1. [Color figure can be viewed at wileyonlinelibrary.com]

Other potential Na^+^ sensors are WAKs. A subdomain of the extracellular domain of WAK1 binds pectin and plays an important role in the sensing and signaling of stresses. This binding is regulated by Ca^2+,^ which maintains the egg‐carton structure of pectin. Monovalent cations such as Na^+^ disrupt the intermolecular Ca^2+^ bridges between PGA polymers. It was hypothesized that WAK1 senses the pectin‐related changes in the cell wall structure; however, the actual mechanism of Na^+^ sensing by WAK1 is still unknown (Decreux and Messiaen 2005). A graphical summary of the possible mechanisms of Na^+^ sensing by various RLKs is presented in Figure 5.

Molecular or electrophysiological information on the salt stress‐dependent roles of RLKs in halophytes is strikingly sparse. The knowledge about the roles of halophytic RLKs in salt stress responses largely comes from genomic or transcriptomic studies. Therefore, comprehensive molecular studies on Na^+^ sensing by RLKs and their participation in salt tolerance are critically needed. Nevertheless, a few studies discerning the roles of RLKs in Na^+^‐related stress have provided a few insights. Halophytes possess multiple homologs of RLK genes as compared to glycophytes. Halophytes have high specificity for binding apoplastic Na^+^ under salt stress. This was evident based on the elevated Na^+^ aggregates in the boiled roots of *T. halophila* as compared to *Arabidopsis* after residual washing. The selective binding of Na^+^ in the cell walls of *T. halophila* can be attributed to Na^+^‐selective binding proteins, but this needs functional validation (Wang et al. 2006). A comparative study of the glycophyte, *Spinacia oleracea* (spinach), and the salt‐accumulating halophyte, Suaeda *salsa* (seepweed), showed no decrease in the pectin structure of the halophyte due to salinity of up to 300 mM NaCl in the cell wall of the roots, but instead the results showed more intact cell wall in *S. salsa* than in *S. oleracea*, which had decreased pectin and reduced cell wall extensibility due to Na^+^ accumulation that disrupted the pectin structure. In addition, *S. salsa* exhibited higher cellulose content, which protects cell wall structural stability in salt stress (Liu et al. 2022b).

Several transcriptomic studies indicate the role of RLKs in Na^+^ perception in halophytes. For instance, the euhalophyte, *Puccinellia tenuiflora* (Alkaligrass), a close relative of wheat (*Triticum aestivum*) and barley (*Hordeum vulgare)*, exhibited four FER orthologs as compared to one in *Arabidopsis*, which were differentially expressed in the root under salt stress. The differential expression of FER was attributed to divergence in the sequence 1 kb upstream of the transcriptional start site in the promoter (Zhang et al. 2020a). In addition, the transcriptomic and qRT‐PCR validation of *CqFER* in *Chenopodium quinoa* (quinoa; a salt accumulator) demonstrated that *CqFER* was significantly upregulated (by 3 to fivefolds) and mediated earlier and faster H_2_O_2_ accumulation, ROS signaling, and greater sensitivity of ROS‐induced Ca^2+^ channels in the tolerant quinoa (Q68) than the salt‐sensitive Q30 accession. The higher membrane integrity and K^+^ retention in the mature root of Q68 was attributed to the constitutively high expression of *FER* even in the absence of salt stress, contributing to the early perception of salt stress in the root (Bazihizina et al. 2021).

The upregulated expressions of *FER*, *THE1*, and *WAK1* in *Nitraria sibirica* Pall (Siberian Nitre Bush; a Na^+^ excluder) indicated their roles in the perception of Na^+^‐induced cell wall damage under 300 mM Na^+^ stress. (Zhang et al. 2023). Genomic analyses of *Chenopodium quinoa* identified 26 *CqCrRLK1L*s and 18 *CqRALF*s. The tissue‐specific transcriptomic profiling revealed various interesting features. For instance, *CqFER*, a homolog of *Arabidopsis AtFER*, is ubiquitously expressed in all tissues. Three *CqCrRLK1L*s (*CqCrRLK1L5*, *CqCrRLK1L9* and *CqCrRLK1L7*) were significantly upregulated under salt stress, and had no homolog in *Arabidopsis*. In addition, an ortholog of *AtRALF22, CqRALF15*, was found to physically interact with and mediate FER internalization, similar to *AtRALF22*, inhibiting root growth in quinoa and *Arabidopsis*, respectively*. The* overexpression of *CqRALF15* promoted bleaching of the leaves under salt stress, implicating its role in salt response (Jiang et al. 2022). In the euhalophyte, *Paspalum vaginatum* (Seashore Paspalum), qPCR analyses showed that an *LLR‐RLK* gene was induced in roots by salt stress after 24 h. Its heterologous expression in yeast enhanced the survival of the transformants in comparison to yeast cells transformed with empty vectors. This implied a positive role in salt tolerance, in contrast to the increased salt sensitivity of *OsGIRL1* (an LRR‐RLK)‐overexpressing rice plants (Chen et al. 2016). *Ipomoea imperati* (beach morning‐glory; a euhalophyte) is a wild relative of sweet potato that had a significantly upregulated *LLR‐RLK* under 600 mM salt stress, implying a possible role of this gene in initiating a sensory response in roots that leads to the transcriptional reprogramming of downstream signaling in ion homeostasis, ROS signaling, and metabolic processes (Luo et al. 2017). *PvWAK3* from *Paspalum vaginatum* (Seashore Paspalum; a salt accumulator) improved salt tolerance in *Arabidopsis* via improving antioxidant activity, upregulating *P5CS1*, and maintaining Na^+^/K^+^ homeostasis (Li et al. 2024).

Several forward genetic screens in glycophytes indicated possible roles of *RLK*s in the modulation of ionic and osmotic stress responses (Ten Hove et al. 2011). However, analogous studies in halophytes are limited. The comparable genomic and transcriptomic studies on halophytes need future functional validation. Certain RLKs, such as FER and WAKs, are conserved in both halophytes and glycophytes, and could perceive Na^+^‐induced signals and induce downstream responses despite significant variations in cell wall structures. The orthologs in halophytes show high basal gene expressions, unique sequence variations, and differences in the cis‐element/promoter sequences, making them strong candidates for transgenic studies on improved salt stress sensing in glycophytes and potential targets for genome editing (Zhang et al. 2020b).

## 7 Conclusion and Future Perspectives

Sodium sensing in halophytes exhibits distinct characteristics as compared to glycophytes. For instance, halophytes such as *S. europaea* (a succulent euhalophyte), when under salt stress, increase their sphingolipid contents, including ceramide (Cer), sphingosine (Sph), sphingosine‐1‐phosphate (S1P), and especially GIPCs, in comparison to glycophytes. These sphingolipids enhance salt tolerance by reducing lipid saturation and improving membrane fluidity, thus enhancing salt tolerance (Yang et al. 2023). The GIPCs in *Arabidopsis* are considered molecular candidates for sensing Na^+^ via direct binding and induce downstream Ca^2+^ signaling that possibly promotes Na^+^ extrusion during salt stress. In addition, NSCCs in salt‐excluding halophytes restrict Na^+^ entry through high K^+^ selectivity, as evident by the voltage‐independent NSCC electrophysiological studies in *T. halophila* and *Arabidopsis* (Volkov et al. 2004). Lower Na^+^ uptake in halophytes, such as *T. halophile*, results in lower energy expenditure required for Na^+^ efflux in comparison to glycophytes, such as *Arabidopsis*, that need to remove excess Na^+^ through energy‐requiring NHX antiporters (Volkov and Amtmann 2006). Moreover, for halophytes such as *Zygophyllales* (salt accumulators), the uptake of Na^+^ is not energy‐intensive, unlike glycophytes that need energy from H^+^‐ATPases for the hyperpolarization‐dependent influx of Na^+^ through the plasma membrane (Ma et al. 2024b). The genomic and transcriptomic studies on halophytes uncovered mostly similar salt stress‐sensing genes. However, these genes might differ in behavior due to different regulatory mechanisms based on their respective environments, in addition to species‐ or tissue‐specific variations among plants (Himabindu et al. 2016). Halophytic *CNGC*, *GLR*, and *RLK* genes have expanded gene families, tandem duplications, higher numbers of and more diverse orthologs and paralogs in comparison to glycophytes. In addition, halophytic genes possess structural modifications that might be attributed to differences in their cis‐regulatory elements, differential gene regulations, and their posttranscriptional or posttranslational modifications in comparison to their counterparts in non‐halophytes.

Previous studies indicated that halophytic genes involved in ion homeostasis, osmoprotectants, antioxidant induction as well as cross‐talk, ectopically expressed in glycophytic plants could mediate crop salt tolerance (Himabindu et al. 2016). For instance, overexpression of an Na^+^/H^+^ tonoplast antiporter from *Nitraria sibirica* (the halophytic shrub) enhanced the salt tolerance of transgenic poplar (Geng et al. 2020). One study showed the overexpression of *PvWAK3* from *Paspalum vaginatum*, a euhalophyte, positively regulated salt tolerance in *Arabidopsis* via an improvement in Na^+^/K^+^ homeostasis, ROS scavenging, and proline biosynthesis (Li et al. 2024). However, more transgenic studies of ectopically expressing Na^+^ sensing‐related genes from halophytes (such as *CNGC*s, *GLR*s, and GIPC‐biosynthetic genes) in glycophytes to improve crop salt tolerance are needed. Presently, most studies focus on manipulating glycophytic *RLK*s to mediate salt tolerance in crops. For instance, the overexpression of rice *OsWAK112* in rice and *Arabidopsis* decreased survival while knocking down *OsWAK112* enhanced survival under salt stress, implicating its negative role in salt tolerance via binding with OsSAMS1/2/3 and inhibiting ethylene production (Lin et al. 2021). In addition, overexpression of *Glycine soja GsSRK*s (*G‐type LecRLK*s) in alfalfa enhanced salt tolerance by improving osmotic adjustment, ROS scavenging, and ion homeostasis (Sun et al. 2018). However, more investigations into the heterologous expression of halophytic *CNGC*s and *GLR*s to improve crop salt tolerance are needed.

Research on halophytes has shown diverse developmental and physiological plasticity (including ion sequestration, osmotic adjustment, glands, and bladders), which suggests their role in salt tolerance. However, a knowledge gap exists in studies determining the exact Na^+^ sensing and perception mechanisms in halophytes. It is not clear whether these putative Na^+^‐sensing genes can be transferred to crops without yield penalties. In addition, the lack of a specific halophyte model system, like the one in glycophytes, hinders a clear understanding of Na^+^ sensing in these plants. Future studies should examine genetically trackable halophytic species to determine and functionally characterize their Na^+^ perception mechanisms and their possible translation to salt tolerance in crops (Van Zelm et al. 2020). Moreover, forward and reverse genetic studies should be conducted in halophytes, such as *Eutrema* or *Salicornia*, to determine the GIPC biosynthesis‐related genes. It will be useful to determine the orthologs of *MOCA1* or *IPUT1* in halophytes, as well as the tandem duplication of the genes involved in GIPC biosynthesis. In addition, sequence comparisons of GIPC biosynthetic genes should be conducted in halophytes to determine the basis of the Na^+^‐specific binding of GIPC during salt stress. Future investigations into the differential regulations, structural modifications, and posttranscriptional/posttranslational modifications of Na^+^‐sensing halophytic genes will further enhance our understanding of Na^+^‐sensing mechanisms in halophytes. For instance, it is imperative to determine the restrictive nature and high K^+^ specificity of the halophytic CNGC as compared to its glycophytic counterparts, given their potential energetic costs. The restriction of Na^+^ influx by CNGC has been shown to enhance crop salt tolerance. For instance, the downregulation of the rice (*Oryza sativa*) *OsCNGC1* in roots increased the salt tolerance of cultivar FL478 (Senadheera et al. 2009). Similarly, *OsCNGC1* downregulation in the Egyptian Yasmine rice cultivar also improved its salt tolerance (Mekawy et al. 2015). The sequence comparison, site mutagenic studies, and molecular and functional validations of halophytic Na^+^‐sensing genes (*CNGC/GLR/RLK*) will provide valuable insights into increasing salt tolerance in crops. Moreover, single‐cell‐specific comparative genomic, transcriptomic, proteomic, and metabolomic studies of glycophytes and their halophytic relatives should be conducted to understand Na^+^ sensing and perception at the cellular as well as the organismal levels.

## Conflicts of Interest

Hon‐Ming Lam is an Editorial Board member of *Plant, Cell & Environment* and a coauthor of this article. To minimize bias, he was excluded from all editorial decision‐making related to the acceptance of this article for publication.

## Supporting information


**Table S1:** Potential cyclic nucleotide cation channels (CNGCs) for sodium ion (Na^+^) sensing in glycophytes under salt stress.
**Table S2:** Potential glutamate receptors (GLRs) for sodium (Na^+^) sensing in glycophytes.
**Table S3:** List of receptor‐like kinases involved in sodium (Na^+^) stress responses and their potential roles in Na^+^ sensing.

## Data Availability

Data sharing not applicable to this article as no datasets were generated or analysed during the current study.
