# Supplementary material for: Deciphering the Sodium Sensing Mechanisms in Glycophytes and Halophytes
Source: Plant Cell Environ. 2025 Sep 1;48(12):8586–603. doi: 10.1111/pce.70128 (PMC12586917; doi:10.1111/pce.70128)
Supplement: Supplementary file 1 — Table S1: Potential cyclic nucleotide cation channels (CNGCs) for sodium ion (Na+) sensing in glycophytes under salt stress. Table S2: Potential glutamate receptors (GLRs) for sodium (Na+) sensing in glycophytes. Table S3: List of receptor‐like kinases involved in sodium (Na+) stress responses and their potential roles in Na+ sensing. [file PCE-48-8586-s001.docx]

**Supplementary Information**

**Table S1.** Potential cyclic nucleotide cation channels (CNGCs) for sodium ion (Na^+^) sensing in glycophytes under salt stress.

| Genes | Plant Species | Functions | References |
| --- | --- | --- | --- |
| *GhCNGC32* and *GhCNGC35* | *Gossypium spp.* | VIGS^[[1]](#footnote-1)^ silencing reduced the salt tolerance of cotton in an abscisic acid (ABA)-dependent way. Displayed a significant stress response. | Lu et al., 2022 |
| *GsCNGC20-d* | *Glycine soja* and *Glycine max* | Overexpression of root-specific *GsCNGC20-d* in soybean and *Arabidopsis* enhanced salt tolerance. Co-overexpression with *GsCDPK29* in soybean greatly improved salt tolerance. | Pi et al., 2023 |
| *BnaCNGC19* | *Brassica napus* | Showed NaCl stress-induced upregulation specifically in roots. | Liu et al., 2021 |
| *ZjCNGC4, ZjCNGC2,* and *ZjCNGC6* | *Ziziphus jujuba* Mill. | *ZjCNGC4* was induced under cold, salt, and alkaline stress, whereas *ZjCNGC2*, 4, and 6 were prominently upregulated in salt stress conditions. | Wang et al., 2020 |
| *SsCNGC1* | *Saccharum spontaneum* | *SsCNGC1* is hypothesized to play a crucial role in stem elongation and maintaining Na^+^ and K^+^ balance. | Zhang et al., 2023 |
| *HvCNGC19* | *Hordeum vulgare* | It has a close relation with *CNGC19* as it shares motif 24 with *Arabidopsis*, suggesting similar expression patterns to *AtCNGC19* under salt stress. | Oranab et al., 2023 |
| *OsCNGC4* and  *OsCNGC11* | *Oryza sativa* | Their ion transport domain possesses motif 24 and it closely mirrors *AtCNGC19.* | Oranab et al., 2023 |
| *HvCNGC2-3* | *Hordeum vulgare* | Exhibited a distinct dual-ion activation, showing a weak voltage-dependent ion current in the co-presence of Na^+^, K^+^, and 8Br-cAMP due to the presence of the distinctive ion selectivity sequence, AQGL. | Mori et al., 2018 |
| *HvCBT1* | *Hordeum vulgare* | Has calmodulin-binding ability, possesses a cyclic nucleotide-binding site, and localizes within the plasma membrane. | Maathuis and Sanders, 2001 |
| *GhCNGC1* and *GhCNGC18* | *Gossypium spp.* | Silencing of these genes enhanced resistance to *Verticillium* wilt and salt stress in plants, stunting growth and development. | Zhao et al., 2022 |
| *GhCNGC12* and *GhCNGC*31 | *Gossypium spp.* | Silencing led to increased sensitivity to salt stress. | Zhao et al., 2022 |
| *GhCNGC25* | *Gossypium spp.* | Showed significantly induced expression under 200 mM NaCl-induced salt stress. | Zhao et al., 2022 |
| *GhCNGC11*,  *15* and *29* | *Gossypium spp.* | Demonstrated a salt stress response analogous to that by *AtCNGC10*. | Zhao et al., 2022 |
| *SmCNGC17* | *Solanum melongena* | Showed elevated expression under salt stress and KEGG^2^ analyses revealed involvement in plant-pathogen interaction. | Shen et al., 2022 |
| Putative *CNGC15* and *CNGC14* | *Vitis vinifera* L. | Possibly involved in ion transport, exhibited significant upregulation under salt stress conditions and remains uncharacterized. | Guan et al., 2018 |
| Putative *CNGC17* | *Vitis vinifera* L. | While uncharacterized, it was downregulated under salt stress and potentially contributes to ion transportation. | Guan et al., 2018 |
| *BRADI2G51836* (CNGC4*)* | *Brachypodium distachyon*(L.) Beauv. | During salt stress, its elevated expression facilitated Na^+^ transport into the cytoplasm. | Guo et al., 2020 |
| *NP974783.2 (CNGC2)* | *Raphanus sativus* L. | Elevated in response to salt stress but lacks functional characterization. | Sun et al., 2016 |
| *W9R5Q9* (*CNGC8)* | *Morus alba* L. | It is a putative cyclic nucleotide-gated ion channel that was downregulated under salt stress. | Wang et al., 2023 |
| *CNGC1* | *Morus alba* L. | Protein expression decreases under salt stress conditions and is involved in ion transport. | Yang and Wu, 2021 |
| *CNGC4* (Si026386m.g) | *Setaria italic*L. | Elevated expression during salt stress suggests a possible involvement in sensing and signaling salt stress. | Pan et al., 2020 |
| *CNGC1*(LOC112697431) and *CNGC4* (LOC112771534) | *Arachis hypogaea* L. | External calcium reduced the expression of these two non-selective ion channel genes during salt stress and potentially reduced Na^+^ uptake. | Dong et al., 2023 |
| *CNGC2* (Ca26987) | *Cicer arietinum* L. | Highly induced under salt stress, possibly involved in ion transport. | Khan et al., 2023 |
| *CNGC12* (Ca17275) | *Cicer arietinum* L. | Reduced expression under salt stress, potentially impacting ion transport. | Khan et al., 2023 |
| *CNGC20* (Ca32080) | *Cicer arietinum* L. | Induced in response to salt stress. | Khan et al., 2023 |
| *CNGC* (MTR_7g012260) | *Medicago sativa* | Showed differential gene expression associated with ion transport after salt treatment, especially for CNGCs integral to Ca^2+^ signal transduction. | Ma et al., 2020 |

**Table S2.** Potential glutamate receptors (GLRs) for sodium (Na^+^) sensing in glycophytes.

| Genes | Plant species | Functions | References |
| --- | --- | --- | --- |
| *OsGLR3. 4* | *Oryza sativa* | Gene mutation resulted in glycine-triggered membrane depolarization and ROS production at the root tip's elongation zone. | Yu et al., 2023 |
| *OsGLR2.1* | *Oryza sativa* | Expression in bacterial assays indicated its ability in facilitating ion uptake and Ca^2+^ signaling. | Ni et al., 2016 |
| *OsGLR3.2* | *Oryza sativa* | Bacterial growth experiments showed *OsGLR3.2* possesses the capability to facilitate ion uptake in bacteria. | Ni et al., 2016 |
| *AtGLR1.4* | *Arabidopsis thaliana* | Plays a role in the membrane depolarization induced by methionine in *Arabidopsis* leaves. | Tapken et al., 2013 |
| *AtGLR3.4* | *Arabidopsis thaliana* | *GLR3.4* cDNA expression in HEK cells revealed a significant ionic current in the presence of amino acids and green fluorescent protein (GFP) with greater permeability for Ca^2+^ than Na^+^. | Vincill et al., 2012 |
| *GLR3.4* | *Arabidopsis thaliana* | *GLR3.4* was activated by asparagine, serine, and glycine in HEK cells and showed weak membrane potential response in *glr3.4* mutant plants. | Stephens et al., 2008 |
| *GLR3.3* | *Arabidopsis thaliana* | *glr3.3* mutant had impaired glutamate-induced membrane depolarization and Ca^2+^ induction, suggesting its role in cation mobility across membranes. | Qi et al., 2006 |
| *AtGluR2* | *Arabidopsis thaliana* | *AtGluR2*-overexpression altered Ca^2+^ utilization and increased Na^+^ and K^+^ sensitivity, suggesting a role in sensing mineral ions | Kim et al., 2001 |
| *GLR* | *Arabidopsis thaliana* | A patch-clamp investigation suggested that glutamate activated the glutamate receptor in root cells, directing voltage-insensitive ionic currents with elevated Ca^2+^ without direct uptake of Na^+^. | Demidchik et al., 2004 |
| *GLR* | *Arabidopsis thaliana* | Glutamate induced membrane depolarization, disrupted cortical microtubules, and reduced root growth within a short timeframe. | Sivaguru et al., 2003 |
| *AtGLR3·2* | *Arabidopsis thaliana* | Overexpression of *AtGLR3·2* led to calcium deficiency symptoms and increased sensitivity to K^+^ and Na^+^. | Kim et al., 2001 |
| *AtGLR3·7* | *Arabidopsis thaliana* | *AtGLR3·7* ectopic expression in *Xenopus* oocytes showed it to be a continuously active, calcium-permeable, non-selective cation channel, not requiring induction by glutamate or other iGluR agonists. | Chen et al., 2021 |
| *AtGLR3.4* | *Arabidopsis thaliana* | Mutation by an animal glutamate receptor antagonist (DNQX) blocked NaCl-induced Ca^2+^ waves, leading to reduced NaCl-triggered Ca^2+^ increase and Na^+^ accumulation. | Cheng et al., 2018 |
| *AtGLR3.7* | *Arabidopsis thaliana* | *AtGLR3.7* ectopic expression in *Xenopus* oocyctes caused the plasma membrane to become permeable to Ba^2+^, Ca^2+^, and Na^+^. | Roy et al., 2008 |
| *AtGLR1.1* and *AtGLR1.4* | *Arabidopsis thaliana* | Introducing the pore domains of *AtGLR1.1* and *AtGLR1.4* into rat *GluR1* and *GluR6* chimeras resulted in the formation of functional channels permeable to K^+^, Na^+^, and Ca^2+^. | Roy et al., 2008 |
| *AtGLR1.1* and *AtGLR3.4* | *Arabidopsis thaliana* | *AtGLR1.1* and *AtGLR3.4* expression in HEK293 cells created homomeric channels for conducting ions. | Price et al., 2013 |
| *GLR3.3* and *GLR3.5* | *Solanum lycopersicum* | *GLR3.3* and *GLR3.5* contributed to electrical signal transduction and activation of jasmonic acid (JA) signaling. | Hu et al., 2021 |
| *IbGLR19* | *Ipomoea batatas* | Salt stress suppressed the expression of *IbGLR19* in the salt-sensitive Lizixiang variety. | Hu et al., 2023 |
| *BraGLR1*  and *BraGLR 9* | *Brassica napus* | *AtGLR3.2*, like *BraGLR1*, was induced under salt stress and interacted with *AtCNGC19, BraGLR1,* and *BraGLR9* to aid in the salt stress response. | Yang et al., 2022 |
| *GmGLR2.5* | *Glycine max* | The significant upregulation of *GmGLR2.5* under salt stress indicated its crucial function in enhancing resistance to salt stress. | Li et al., 2023 |
| *AtGLR3.7* | *Arabidopsis thaliana* | *AtGLR3.7* interacted with 14-3-3 omega to regulate the salt stress response. The *atglr3.7-2* mutant displayed heightened sensitivity to salt stress, whereas overexpression of *AtGLR3.7* had the opposite effect. | Wang et al., 2019 |

**Table S3.** List of receptor-like kinases (RLKs) involved in sodium (Na^+^) stress responses and their potential roles in Na^+^ sensing.

| Receptor-Like Kinase (RLK) family | Genes | Functions and pathways | Plant species | References |
| --- | --- | --- | --- | --- |
| *Catharanthus roseus* RLK1-like kinases (CrRLK1Ls) | *HERK1* | *HERK1*, along with *Feronia* and *Thesus1*, reduces salt stress by regulating pectin modifications | *Arabidopsis thaliana* | Gigli-Bisceglia et al., 2020 |
|  | *THE1* | A receptor for RALF34, controls cell wall integrity and regulates lateral root initiation | *Arabidopsis thaliana* | Hématy et al., 2007; Gonneau et al., 2018 |
| Leucine-rich repeat-receptor-like kinases (LRR-RLKs) | *OsSIK2* | Participates in senescence and abiotic stress tolerance and is highly induced by NaCl, cold, dark, drought, and abscisic acid (ABA). Salt, darkness, and drought activate downstream *Pathogenesis-related* (*PR*) genes with full-length *OsSIK2* and DREB-like genes through the truncated *OsSIK2.* | *Oryza sativa* | Chen et al., 2013 |
|  | *OsSIK1* | Its overexpression promotes salt tolerance by reducing ion leakage to maintain membrane integrity and by decreasing the malondialdehyde (MDA) content. | *Oryza sativa* | Ouyang et al., 2010 |
|  | *PEPR1* | NaCl strongly induces *AtPep3* to bind to the PEPR1 receptor to enhance salt tolerance. The *AtPep3/AT13* peptide might help in the mitigation of Na^+^-induced ionic stress. | *Arabidopsis thaliana* | Nakaminami et al., 2018 |
|  | *RPK1* | Senses environmental stresses such as salt stress via ABA-dependent signaling. | *Arabidopsis thaliana* | Hong et al., 1997 |
|  | *AtRPK1* and *OsRPK1* | Overexpression of two *RPK1*s in *A. thaliana* lowered salt tolerance by enhancing membrane permeability via increasing Na^+^ influx into the cell, blocking the SOS pathway, and lowering *P5CS1* expression. In contrast, silencing of *RPK1* increased salt tolerance. | *Arabidopsis thaliana*, *Oryza sativa* | Shi et al., 2014 |
|  | *MIK2/LRR-KISS* | Loss of *MIK2* function is thought to impair(Cell wall integrity) CWI sensing, leading to local changes in cell wall composition, resulting in root skewing, and increased sensitivity to salt stress. | *Arabidopsis thaliana* | Van der Does et al., 2017 |
|  | *TaPRK2697* | Enhances tolerance to salt when the TaSR (*Triticum aestivum* Salt Responsive Gene) ligand interacts with the extracellular domain of TaPRK2697 and forms a complex that promotes kinase receptor phosphorylation and triggers the internal stress signal cascades. The overexpression of this gene improves Na^+^ efflux and corresponds to signals for stress resistance. | *Triticum aestivum* | Ma et al., 2016 |
| Lectin domain-containing receptor-like kinases (LecRLKs) | *PsLecRLK* | Enhances salt stress tolerance by reducing Na^+^ influx across prokaryotic cell membranes and plant roots and shoots, and increasing ROS production. | *Pisum sativum* | Joshi et al., 2010 |
|  | *AtLecRK2* | Highly induced by salt stress and ethylene, and may be involved in the transmission of extracellular Na^+^ signals to intracellular signals through its kinase (serine\threonine) activity in an ethylene-dependent manner. | *Arabidopsis thaliana* | He et al., 2004 |
|  | *GsSRK* | Regulates salt stress responses and plant architecture by enhancing ROS scavenging, osmotic regulation, and ion homeostasis, with *GsSRK*-overexpressing transgenic alfalfa showing lower Na^+^ and slightly elevated K^+^ than wild-type. | *Medicago sativa* | Sun et al., 2018 |
|  | *OsLecRLK* | Senses salt stress via lectin receptor and activates kinase domain-mediated signaling, promotes Na^+^ exclusion through SOS1 pathway leading to reduced ROS, toxic ions, and malondialdehyde, increased proline, and ROS scavengers. | *Oryza sativa* | Passricha et al., 2020 |
|  | *SIT1* | Activated by NaCl, drives salt sensitivity through *MPK3/6* activation and promotes ethylene signaling and ROS accumulation, causing growth inhibition and plant death under salt stress. | *Oryza sativa* | Li et al., 201) |
|  | *OsJRL* | Upregulated in response to salt, heat, and drought stresses, *OsJRL* performs a role in stress signal transduction and cell protection. Its overexpression in rice and *E. coli* enhanced salinity tolerance and increased the expression of stress-related genes and genes involved in Na^+^ transport. | *Oryza sativa* | He et al., 2017 |
| Wall-associated kinases (WAKs) | *AtWAKL4* | Permeable and hypersensitive to Na^+^, K^+^, Cu2^+^, Ni^2+^, and Zn^2+^, it plays a role in mineral ion responses. Its hypersensitivity suggests that WAKL4 may be involved in sensing minerals. | *Arabidopsis thaliana* | Hou et al., 2005 |
|  | *AtWAK1* | The extracellular domain of WAK1 binds to pectin components in the presence of Ca^2+^ *in vitro* and at high NaCl concentrations. Depolymerization and methyl esterification of pectin can inhibit Ca^2+^-induced bridges of pectin and ultimately the WAK1-pectin fraction connection | *Arabidopsis thaliana* | Decreux and Messiaen, 2005 |
|  | *AtWAK2* | WAK2 is involved in pectin sensing in response to various external stimuli, by activating MAPK3 activity. | *Arabidopsis thaliana* | Kohorn et al., 2009 |
|  | *AtWAK2* | WAK2 interacts with pectin in the cell wall and may play a role in sensing environmental factors such as salt. The reduction in vacuolar invertase activity due to WAK2 maintains turgor pressure in growing cells. | *Arabidopsis thaliana* | Kohorn et al., 2006 |
|  | *SlWAK1* | *SlWAK1* is required to re-establish osmotic homeostasis and for sucrose transport balance between leaf sources and root sinks. The *Slwak1* mutant has reduction in shoot growth despite decrease in Na^+^ toxicity | *Solanum*  *lycopersicum* | Meco et al., 2020 |
|  | *OsWAK11* | Aluminum, sodium, and copper regulate *OsWAK11* expression. It shows strong upregulation in response to metals and wounding. | *Oryza sativa* | Hu et al., 2014 |
| Cysteine-rich repeat domain-containing receptor-like kinases (CRKs) | *CRK2* | It promotes salt tolerance in *Arabidopsis* by enhancing callose deposition and plasmodesmal permeability upon salt stress during germination. | *Arabidopsis thaliana* | Hunter et al., 2019 |
| Annexins | *GhANN8b* | It influences salinity responses by reversely modulating Na^+^ and Ca^2+^ fluxes and interacts with cotton phosphatase *GhDsPTP3a*. | *Gossypium*  *herbaceum* | Mu et al., 2019 |
|  | *GhANN1* | It produces a Ca^2+^-dependent protein that stimulates the upregulation of genes related to Na^+^ and K^+^ transport in roots. | *Gossypium herbaceum* | Zhang et al., 2021 |
| Cytoplasmic receptor-like kinases | *STRK1* | Its overexpression resulted in a high tolerance to salt with a decreased Na^+^/K^+^ ratio, suggesting its partial involvement in the SOS pathway by restraining Na^+^ accumulation. | *Oryza sativa* | Zhou et al., 2018 |

**References**

CHEN, L.-J., WURIYANGHAN, H., ZHANG, Y.-Q., DUAN, K.-X., CHEN, H.-W., LI, Q.-T., LU, X., HE, S.-J., MA, B. & ZHANG, W.-K. 2013. An S-domain receptor-like kinase, OsSIK2, confers abiotic stress tolerance and delays dark-induced leaf senescence in rice. *Plant physiology,* 163**,** 1752-1765.

CHEN, P.-Y., HSU, C.-Y., LEE, C.-E. & CHANG, I.-F. 2021. Arabidopsis glutamate receptor GLR3. 7 is involved in abscisic acid response. *Plant Signaling & Behavior,* 16**,** 1997513.

CHENG, Y., ZHANG, X., SUN, T., TIAN, Q. & ZHANG, W.-H. 2018. Glutamate receptor homolog3. 4 is involved in regulation of seed germination under salt stress in Arabidopsis. *Plant and Cell Physiology,* 59**,** 978-988.

DECREUX, A. & MESSIAEN, J. 2005. Wall-associated kinase WAK1 interacts with cell wall pectins in a calcium-induced conformation. *Plant and Cell Physiology,* 46**,** 268-278.

DEMIDCHIK, V., ESSAH, P. A. & TESTER, M. 2004. Glutamate activates cation currents in the plasma membrane of Arabidopsis root cells. *Planta,* 219**,** 167-175.

DONG, X., GAO, Y., BAO, X., WANG, R., MA, X., ZHANG, H., LIU, Y., JIN, L. & LIN, G. 2023. Multi-Omics Revealed Peanut Root Metabolism Regulated by Exogenous Calcium under Salt Stress. *Plants,* 12**,** 3130.

GIGLI-BISCEGLIA, N., VAN ZELM, E., HUO, W., LAMERS, J. & TESTERINK, C. 2020. Salinity stress-induced modification of pectin activates stress signaling pathways and requires HERK/THE and FER to attenuate the response. *BioRxiv***,** 2020.12. 18.423458.

GONNEAU, M., DESPREZ, T., MARTIN, M., DOBLAS, V. G., BACETE, L., MIART, F., SORMANI, R., HÉMATY, K., RENOU, J. & LANDREIN, B. 2018. Receptor kinase THESEUS1 is a rapid alkalinization factor 34 receptor in Arabidopsis. *Current Biology,* 28**,** 2452-2458. e4.

GUAN, L., HAIDER, M. S., KHAN, N., NASIM, M., JIU, S., FIAZ, M., ZHU, X., ZHANG, K. & FANG, J. 2018. Transcriptome sequence analysis elaborates a complex defensive mechanism of grapevine (Vitis vinifera L.) in response to salt stress. *International journal of molecular sciences,* 19**,** 4019.

GUO, X., WANG, Q., LIU, Y., ZHANG, X., ZHANG, L. & FAN, S. 2020. Screening of Salt Stress Responsive Genes in Brachypodium distachyon (L.) Beauv. by Transcriptome Analysis. *Plants,* 9**,** 1522.

HE, X.-J., ZHANG, Z.-G., YAN, D.-Q., ZHANG, J.-S. & CHEN, S.-Y. 2004. A salt-responsive receptor-like kinase gene regulated by the ethylene signaling pathway encodes a plasma membrane serine/threonine kinase. *Theoretical and Applied Genetics,* 109**,** 377-383.

HE, X., LI, L., XU, H., XI, J., CAO, X., XU, H., RONG, S., DONG, Y., WANG, C. & CHEN, R. 2017. A rice jacalin‐related mannose‐binding lectin gene, Os JRL, enhances Escherichia coli viability under high salinity stress and improves salinity tolerance of rice. *Plant Biology,* 19**,** 257-267.

HÉMATY, K., SADO, P.-E., VAN TUINEN, A., ROCHANGE, S., DESNOS, T., BALZERGUE, S., PELLETIER, S., RENOU, J.-P. & HÖFTE, H. 2007. A receptor-like kinase mediates the response of Arabidopsis cells to the inhibition of cellulose synthesis. *Current Biology,* 17**,** 922-931.

HONG, S. W., JON, J. H., KWAK, J. M. & NAM, H. G. 1997. Identification of a receptor-like protein kinase gene rapidly induced by abscisic acid, dehydration, high salt, and cold treatments in Arabidopsis thaliana. *Plant physiology,* 113**,** 1203-1212.

HOU, X., TONG, H., SELBY, J., DEWITT, J., PENG, X. & HE, Z.-H. 2005. Involvement of a cell wall-associated kinase, WAKL4, in Arabidopsis mineral responses. *Plant physiology,* 139**,** 1704-1716.

HU, C., DUAN, S., ZHOU, J. & YU, J. 2021. Characteristics of herbivory/wound-elicited electrical signal transduction in tomato. *Frontiers of Agricultural Science and Engineering,* 8**,** 292-301.

HU, W., LV, Y., LEI, W., LI, X., CHEN, Y., ZHENG, L., XIA, Y. & SHEN, Z. 2014. Cloning and characterization of the Oryza sativa wall-associated kinase gene OsWAK11 and its transcriptional response to abiotic stresses. *Plant and soil,* 384**,** 335-346.

HU, Y., DAI, Z., HUANG, J., HAN, M., WANG, Z., JIAO, W., GAO, Z., LIU, X., LIU, L. & MA, Z. 2023. Genome-wide identification and expression analysis of the glutamate receptor gene family in sweet potato and its two diploid relatives. *Frontiers in Plant Science,* 14**,** 1255805.

HUNTER, K., KIMURA, S., ROKKA, A., TRAN, H. C., TOYOTA, M., KUKKONEN, J. P. & WRZACZEK, M. 2019. CRK2 enhances salt tolerance by regulating callose deposition in connection with PLD α 1. *Plant Physiology,* 180**,** 2004-2021.

JOSHI, A., DANG, H. Q., VAID, N. & TUTEJA, N. 2010. Pea lectin receptor-like kinase promotes high salinity stress tolerance in bacteria and expresses in response to stress in planta. *Glycoconjugate journal,* 27**,** 133-150.

KHAN, H. A., SHARMA, N., SIDDIQUE, K. H. M., COLMER, T. D., SUTTON, T. & BAUMANN, U. 2023. Comparative transcriptome analysis reveals molecular regulation of salt tolerance in two contrasting chickpea genotypes. *Frontiers in Plant Science,* 14.

KIM, S. A., KWAK, J., JAE, S.-K., WANG, M.-H. & NAM, H. 2001. Overexpression of the AtGluR2 Gene Encoding anArabidopsis Homolog of Mammalian Glutamate Receptors Impairs CalciumUtilization and Sensitivity to Ionic Stress in TransgenicPlants. *Plant and Cell Physiology,* 42**,** 74-84.

KOHORN, B. D., JOHANSEN, S., SHISHIDO, A., TODOROVA, T., MARTINEZ, R., DEFEO, E. & OBREGON, P. 2009. Pectin activation of MAP kinase and gene expression is WAK2 dependent. *The Plant Journal,* 60**,** 974-982.

KOHORN, B. D., KOBAYASHI, M., JOHANSEN, S., RIESE, J., HUANG, L. F., KOCH, K., FU, S., DOTSON, A. & BYERS, N. 2006. An Arabidopsis cell wall‐associated kinase required for invertase activity and cell growth. *The Plant Journal,* 46**,** 307-316.

LI, C.-H., WANG, G., ZHAO, J.-L., ZHANG, L.-Q., AI, L.-F., HAN, Y.-F., SUN, D.-Y., ZHANG, S.-W. & SUN, Y. 2014. The Receptor-Like Kinase SIT1 Mediates Salt Sensitivity by Activating MAPK3/6 and Regulating Ethylene Homeostasis in Rice  *The Plant Cell,* 26**,** 2538-2553.

LI, X., ZHU, T., WANG, X. & ZHU, M. 2023. Genome-wide identification of glutamate receptor-like gene family in soybean. *Heliyon,* 9.

LIU, W., KANG, Y., REN, R., LIU, Y., LI, W., XIE, P., LIAO, L., WANG, W., QIAN, L. & GUAN, M. 2021. Identification and expression analysis of BnaCNGC family gene in the response to phytohormones, abiotic and biotic stresses in Brassica napus. *Journal of Plant Interactions,* 16**,** 575-586.

LU, Z., YIN, G., CHAI, M., SUN, L., WEI, H., CHEN, J., YANG, Y., FU, X. & LI, S. 2022. Systematic analysis of CNGCs in cotton and the positive role of GhCNGC32 and GhCNGC35 in salt tolerance. *BMC genomics,* 23**,** 560.

MA, J., WANG, Y. & LI, J. 2020. Global identification and analysis of microRNAs involved in salt stress responses in two alfalfa (Medicago sativa ‘Millennium’) lines. *Canadian Journal of Plant Science,* 100**,** 445-455.

MA, X. L., CUI, W. N., ZHAO, Q., ZHAO, J., HOU, X. N., LI, D. Y., CHEN, Z. L., SHEN, Y. Z. & HUANG, Z. J. 2016. Functional study of a salt‐inducible TaSR gene in Triticum aestivum. *Physiologia plantarum,* 156**,** 40-53.

MAATHUIS, F. J. & SANDERS, D. 2001. Sodium uptake in Arabidopsis roots is regulated by cyclic nucleotides. *Plant Physiology,* 127**,** 1617-1625.

MECO, V., EGEA, I., ORTÍZ-ATIENZA, A., DREVENSEK, S., ESCH, E., YUSTE-LISBONA, F. J., BARNECHE, F., VRIEZEN, W., BOLARIN, M. C. & LOZANO, R. 2020. The salt sensitivity induced by disruption of cell wall-associated kinase 1 (SlWAK1) tomato gene is linked to altered osmotic and metabolic homeostasis. *International Journal of Molecular Sciences,* 21**,** 6308.

MORI, I. C., NOBUKIYO, Y., NAKAHARA, Y., SHIBASAKA, M., FURUICHI, T. & KATSUHARA, M. 2018. A cyclic nucleotide-gated channel, HvCNGC2-3, is activated by the co-presence of Na+ and K+ and permeable to Na+ and K+ non-selectively. *Plants,* 7**,** 61.

MU, C., ZHOU, L., SHAN, L., LI, F. & LI, Z. 2019. Phosphatase GhDs PTP 3a interacts with annexin protein Gh ANN 8b to reversely regulate salt tolerance in cotton (Gossypium spp.). *New Phytologist,* 223**,** 1856-1872.

NAKAMINAMI, K., OKAMOTO, M., HIGUCHI-TAKEUCHI, M., YOSHIZUMI, T., YAMAGUCHI, Y., FUKAO, Y., SHIMIZU, M., OHASHI, C., TANAKA, M. & MATSUI, M. 2018. AtPep3 is a hormone-like peptide that plays a role in the salinity stress tolerance of plants. *Proceedings of the National Academy of Sciences,* 115**,** 5810-5815.

NI, J., YU, Z., DU, G., ZHANG, Y., TAYLOR, J. L., SHEN, C., XU, J., LIU, X., WANG, Y. & WU, Y. 2016. Heterologous expression and functional analysis of rice GLUTAMATE RECEPTOR-LIKE family indicates its role in glutamate triggered calcium flux in rice roots. *Rice,* 9**,** 1-14.

ORANAB, S., GHAFFAR, A., AHMAD, A., PASHA, M., MUNIR, B., ARIF, S., ISHAQ, S., MAHFOOZ, S., KOUSAR, R. & ZAKIA, S. 2023. Genome-wide analysis of cyclic nucleotide-gated ion channels (CNGCS) of Arabidopsis thaliana under abiotic stresses.

OUYANG, S.-Q., LIU, Y.-F., LIU, P., LEI, G., HE, S.-J., MA, B., ZHANG, W.-K., ZHANG, J.-S. & CHEN, S.-Y. 2010. Receptor-like kinase OsSIK1 improves drought and salt stress tolerance in rice (Oryza sativa) plants. *The Plant Journal,* 62**,** 316-329.

PAN, J., LI, Z., DAI, S., DING, H., WANG, Q., LI, X., DING, G., WANG, P., GUAN, Y. & LIU, W. 2020. Integrative analyses of transcriptomics and metabolomics upon seed germination of foxtail millet in response to salinity. *Scientific reports,* 10**,** 13660.

PASSRICHA, N., SAIFI, S. K., KHARB, P. & TUTEJA, N. 2020. Rice lectin receptor‐like kinase provides salinity tolerance by ion homeostasis. *Biotechnology and Bioengineering,* 117**,** 498-510.

PI, B., LIU, X., HUANG, Q., ZHANG, T. & YU, B. 2023. Comparative transcriptomic analysis of Glycine soja and G. max and functional identification of GsCNGC20-d interacted with GsCDPK29 under salt stress. *Environmental and Experimental Botany,* 206**,** 105185.

PRICE, M. B., KONG, D. & OKUMOTO, S. 2013. Inter-subunit interactions between glutamate-like receptors in Arabidopsis. *Plant signaling & behavior,* 8**,** e27034.

QI, Z., STEPHENS, N. R. & SPALDING, E. P. 2006. Calcium entry mediated by GLR3. 3, an Arabidopsis glutamate receptor with a broad agonist profile. *Plant physiology,* 142**,** 963-971.

ROY, S., GILLIHAM, M., BERGER, B., ESSAH, P., CHEFFINGS, C., MILLER, A., DAVENPORT, R., LIU, L. H., SKYNNER, M. & DAVIES, J. 2008. Investigating glutamate receptor‐like gene co‐expression in Arabidopsis thaliana. *Plant, cell & environment,* 31**,** 861-871.

SHEN, L., ZHAO, E., LIU, R. & YANG, X. 2022. Transcriptome analysis of eggplant under salt stress: AP2/ERF transcription factor SmERF1 acts as a positive regulator of salt stress. *Plants,* 11**,** 2205.

SHI, C.-C., FENG, C.-C., YANG, M.-M., LI, J.-L., LI, X.-X., ZHAO, B.-C., HUANG, Z.-J. & GE, R.-C. 2014. Overexpression of the receptor-like protein kinase genes AtRPK1 and OsRPK1 reduces the salt tolerance of Arabidopsis thaliana. *Plant Science,* 217**,** 63-70.

SIVAGURU, M., PIKE, S., GASSMANN, W. & BASKIN, T. I. 2003. Aluminum rapidly depolymerizes cortical microtubules and depolarizes the plasma membrane: evidence that these responses are mediated by a glutamate receptor. *Plant and Cell Physiology,* 44**,** 667-675.

STEPHENS, N. R., QI, Z. & SPALDING, E. P. 2008. Glutamate receptor subtypes evidenced by differences in desensitization and dependence on the GLR3. 3 and GLR3. 4 genes. *Plant Physiology,* 146**,** 529.

SUN, M., QIAN, X., CHEN, C., CHENG, S., JIA, B., ZHU, Y. & SUN, X. 2018. Ectopic expression of GsSRK in Medicago sativa reveals its involvement in plant architecture and salt stress responses. *Frontiers in Plant Science,* 9**,** 226.

SUN, X., XU, L., WANG, Y., LUO, X., ZHU, X., KINUTHIA, K. B., NIE, S., FENG, H., LI, C. & LIU, L. 2016. Transcriptome-based gene expression profiling identifies differentially expressed genes critical for salt stress response in radish (Raphanus sativus L.). *Plant cell reports,* 35**,** 329-346.

TAPKEN, D., ANSCHÜTZ, U., LIU, L.-H., HUELSKEN, T., SEEBOHM, G., BECKER, D. & HOLLMANN, M. 2013. A Plant Homolog of Animal Glutamate Receptors Is an Ion Channel Gated by Multiple Hydrophobic Amino Acids. *Science Signaling,* 6**,** ra47-ra47.

VAN DER DOES, D., BOUTROT, F., ENGELSDORF, T., RHODES, J., MCKENNA, J. F., VERNHETTES, S., KOEVOETS, I., TINTOR, N., VEERABAGU, M. & MIEDES, E. 2017. The Arabidopsis leucine-rich repeat receptor kinase MIK2/LRR-KISS connects cell wall integrity sensing, root growth and response to abiotic and biotic stresses. *PLoS genetics,* 13**,** e1006832.

VINCILL, E. D., BIECK, A. M. & SPALDING, E. P. 2012. Ca2+ conduction by an amino acid-gated ion channel related to glutamate receptors. *Plant physiology,* 159**,** 40-46.

WANG, L., LI, M., LIU, Z., DAI, L., ZHANG, M., WANG, L., ZHAO, J. & LIU, M. 2020. Genome-wide identification of CNGC genes in Chinese jujube (Ziziphus jujuba Mill.) and ZjCNGC2 mediated signalling cascades in response to cold stress. *BMC genomics,* 21**,** 1-16.

WANG, P.-H., LEE, C.-E., LIN, Y.-S., LEE, M.-H., CHEN, P.-Y., CHANG, H.-C. & CHANG, I.-F. 2019. The glutamate receptor-like protein GLR3. 7 interacts with 14-3-3ω and participates in salt stress response in Arabidopsis thaliana. *Frontiers in Plant Science,* 10**,** 1169.

WANG, Y., JIANG, W., CHENG, J., GUO, W., LI, Y. & LI, C. 2023. Physiological and proteomic analysis of seed germination under salt stress in mulberry. *Frontiers in Bioscience-Landmark,* 28**,** 49.

YANG, J. & WU, Y. 2021. Proteomic Analysis: Explosive Salt Accumulation in Leaves of Morus alba L. under Salt Stress. *Forests,* 12**,** 1384.

YANG, L., ZHAO, Y., WU, X., ZHANG, Y., FU, Y., DUAN, Q., MA, W. & HUANG, J. 2022. Genome-wide identification and expression analysis of BraGLRs reveal their potential roles in abiotic stress tolerance and sexual reproduction. *Cells,* 11**,** 3729.

YU, B., SUN, Y., JIN, X., XIE, Z., LI, X. & HUANG, J. 2023. Rice glutamate receptor-like channel OsGLR3.4 modulates the root tropism growth towards amino acids via plasma membrane depolarization and ROS generation. *Environmental and Experimental Botany,* 205**,** 105146.

ZHANG, D., LI, J., NIU, X., DENG, C., SONG, X., LI, W., CHENG, Z., ZHANG, B. & GUO, W. 2021. GhANN1 modulates the salinity tolerance by regulating ABA biosynthesis, ion homeostasis and phenylpropanoid pathway in cotton. *Environmental and Experimental Botany,* 185**,** 104427.

ZHANG, N., LIN, H., ZENG, Q., FU, D., GAO, X., WU, J., FENG, X., WANG, Q., LING, Q. & WU, Z. 2023. Genome-wide identification and expression analysis of the cyclic nucleotide-gated ion channel (CNGC) gene family in Saccharum spontaneum. *BMC genomics,* 24**,** 281.

ZHAO, J., PENG, S., CUI, H., LI, P., LI, T., LIU, L., ZHANG, H., TIAN, Z., SHANG, H. & XU, R. 2022. Dynamic expression, differential regulation and functional diversity of the CNGC family genes in cotton. *International Journal of Molecular Sciences,* 23**,** 2041.

ZHOU, Y.-B., LIU, C., TANG, D.-Y., YAN, L., WANG, D., YANG, Y.-Z., GUI, J.-S., ZHAO, X.-Y., LI, L.-G. & TANG, X.-D. 2018. The receptor-like cytoplasmic kinase STRK1 phosphorylates and activates CatC, thereby regulating H2O2 homeostasis and improving salt tolerance in rice. *The Plant Cell,* 30**,** 1100-1118.

1. [↑](#footnote-ref-1)
